# The change of drug utilization in China’s public healthcare institutions under the “4 + 7” centralized drug procurement policy: Evidence from a natural experiment in China

**DOI:** 10.3389/fphar.2022.923209

**Published:** 2022-08-23

**Authors:** Jiancheng Lu, Hongfei Long, Yuan Shen, Jing Wang, Xin Geng, Ying Yang, Zongfu Mao, Jinghua Li

**Affiliations:** ^1^ School of Public Health, Jilin University, Changchun, China; ^2^ Department of Drug Information Management, Statistical Information Center, National Health Commission of the People’s Republic of China, Beijing, China; ^3^ Dong Fureng Economic and Social Development School, Wuhan University, Wuhan, China; ^4^ Global Health Institute, Wuhan University, Wuhan, China; ^5^ School of Public Health, Wuhan University, Wuhan, China

**Keywords:** drug utilization, drug use, centralized procurement, pooled procurement, China

## Abstract

**Background:** Improving drug accessibility and rational drug use are major challenges for China’s healthcare reform. In 2018, the Chinese government introduced a novel nationwide policy of centralized drug procurement for off-patent drugs, focusing on improving drug utilization patterns of public medical institutions.

**Objective:** To estimate the impacts of the Chinese centralized drug procurement policy (the so-called “4 + 7” policy) on drug utilization in public medical institutions.

**Methods:** A retrospective natural experimental design and difference-in-difference method were applied using cross-region data extracted from the national procurement database. Eleven “4 + 7” pilot cities (intervention group) and eleven non-pilot provinces (control group) were matched. In addition, “4 + 7” policy-related drugs (*n* = 116) were selected as study samples, including 25 drugs in the 4 + 7” procurement List (“4 + 7” List drugs) and their alternative drugs (*n* = 91) that have not yet been covered by centralized procurement policy. Then, the “4 + 7” List drugs were divided into bid-winning and non-winning drugs according to the bidding results, and they were sorted into generic and original drugs. Defined daily dose (DDD) was used to standardize the quantity of drugs used.

**Results:** In the 1-year procurement period, the overall completion rate of agreed procurement volume reached 191.4% in pilot cities. Owing to policy impact, the consumption increased by 405.31% in bid-winning drugs (*β* = 1.62, *p* < 0.001) and decreased by 62.28% (*β* = −0.98, *p* < 0.001) in non-winning drugs. The overall use proportion of bid-winning drugs increased from 17.03% to 73.61% with statistical significance (*β* = 1.48, *p* < 0.001), and increments were also detected in all healthcare settings, regions, and anatomical therapeutic chemical (ATC) categories (all *p*-values < 0.05). Generics and originators were detected with 67.53% increment (*β* = 0.52, *p* < 0.001) and 26.88% drop (*β* = −0.31, *p* = 0.006) in consume volume. The use proportion of generics increased from 59.23% to 78.44% with significance (*β* = 0.24, *p* < 0.001), as well as in tertiary hospitals (*β* = 0.31), secondary hospitals (*β* = 0.23), and primary healthcare centers (*β* = 0.11) (all *p*-values < 0.001). The use proportion of relatively quality-guaranteed drugs (i.e. bid-winning and original drugs) increased from 56.69% to 93.61% with significance (*β* = 0.61, *p* < 0.001), and similar increments were also detected in all healthcare settings, regions, and ATC categories (all *p*-values < 0.05).

**Conclusion:** Healthcare providers demonstrated good compliance with the “4 + 7” policy in completing contracted procurement volume. Centralized drug procurement policy promoted drug consumption gradually concentrated on bid-winning drugs, generic drugs, and more importantly, quality-guaranteed drugs.

## Introduction

In China, obtaining access to appropriate medicines at affordable prices is still a pressing healthcare issue for 1.4 billion Chinese citizens (Fu, 2017; Shi, 2020). Medical institutions are the primary setting of patients’ drug use in China, and more than 80% of consumed drugs reached patients through the medical institution channel (other than retail pharmacies). However, it is known that a general benefit connection existed between hospitals and pharmaceutical enterprises, which lead to induced demands and made physicians exhibit strong financial motivation to prescribe more expensive drugs (Yip et al., 2012; Zeng et al., 2014). Even after the abolition of hospital drug markups, the benefit connection has not been completely severed (Yi et al., 2015). In this context, drug spending in China constantly increased at a growth rate of about 15% (Zeng, 2013; Zeng et al., 2014), and from 2010 to 2018, it accounted for 30–40% of the total health expenditures (NHC, 2020). More worryingly, no effective incentive is found for rational drug production or drug prescribing under the policy context (Hu and Mossialos, 2016).

In 2018, the Chinese government introduced the implementation of national centralized drug procurement of off-patent drugs, to explore the market-oriented drug price formation mechanism. Except for the primary purpose of price reduction by improving competition, the centralized drug procurement policy bears the mission to cut off the space of drug rebates and lead the standardized clinical medication (General Office of the State Council, 2019a). Through the policy measures of “guarantee of use” (Yang et al., 2021a), physicians are encouraged to give priority to prescribing bid-winning ones among products that share an International Nonproprietary Name (INN). The first round pilot of the centralized procurement policy was implemented in four municipalities (Beijing, Tianjin, Shanghai, and Chongqing) and seven subprovincial cities (Shenyang, Dalian, Xiamen, Guangzhou, Shenzhen, Chengdu, and Xi’an) in mainland China, thus known as the “4 + 7” pilot, with 25 drug INNs procured (Joint Procurement Office, 2018a).

Previous studies revealed fruitful evidence on drug utilization change after the implementation of “4 + 7” centralized drug procurement: for instance, the prominently increased use of bid-winning drugs after policy intervention (Yang et al., 2021a; Yang et al., 2021b; Chen et al., 2021; Wen et al., 2021; Wang et al., 2022). Besides, Wang et al. (2022), Yang et al. (2022a), and Xie et al. (2021) revealed the increase in substitution rate of generic drugs based on the descriptive comparison before and after “4 + 7” policy. Wang Y. et al. (2021) reported a reduction in the irrational utilization rate of antiplatelet drugs from 10.54 to 1.60% in one hospital. He et al. (2021) surveyed related physicians and patients, and they reported their generally good recognition and acceptance of bid-winning drugs.

However, the abovementioned research findings were mainly derived from descriptive analysis derived from limited sampled data, which might restrict causal inference and the extrapolation of research findings. In addition, in China, drug utilization condition varies between different healthcare settings, geographical regions, drug therapeutic categories, etc. (Chinese Pharmaceutical Association, 2020; Yang et al., 2022b). In light of this, a need exists for comprehensive empirical studies to systematically landscape the changing patterns in drug utilization under the “4 + 7” policy implementation in different regions, healthcare settings, and INN categories. In the present study, we conducted a natural experiment using the national centralized drug procurement data in China to estimate the changing pattern of drug utilization in the context of the “4 + 7” centralized drug procurement pilot implementation.

## Research framework

### Intervention elements

As a pharmaceutical reform with multidimensional target attributes and multiple intervention measures, the policy practices of the national centralized procurement policy has been systematically introduced by previous scholars (Yang et al., 2021a; Chang, 2021; Hu, 2021; Yuan et al., 2021). In this study, we focus on the policy measures mostly directly related to medical institutions’ drug utilization, which are systematically elaborated as follows:

1) *Drug selection and the determination of centralized procurement List*. Drug INNs with more historical clinical consumption as well as high historical procurement costs were selected as target procurement drugs to conduct centralized bidding (General Office of the State Council, 2019a).
2) *Eligibility criteria for bidding in terms of drug quality*. The Generic Consistency Evaluation (GCE), which was introduced by the National Medical Products Administration (NMPA) to ensure the quality of Chinese generic drugs, is equivalent to their counterpart originators and was set as the eligibility criteria for some particular drugs to be able to participate in bidding activities. In this regard, only the generic drugs passed the GCE, and original drugs were considered eligible to participate in the “4 + 7” centralized procurement (General Office of the State Council, 2019a).
3) *Ancillary supporting policy measures by healthcare commissions*. The National Health Commission (NHC) introduced the supporting policy to encourage the priority use of bid-winning drugs in public medical institutions. To ensure the completion of the contracted procurement volume, a standardized assessment mechanism has been established (NHC, 2019a; NHC, 2019b).
4) *Ancillary supporting policy measures by the healthcare insurance sector*. The National Healthcare Security Administration (NHSA) launched a supporting policy to reward behaviors to save medical insurance funds by using low-priced bid-winning drugs (NHSA, 2019a).

### Analytical framework

**FIGURE 1:**
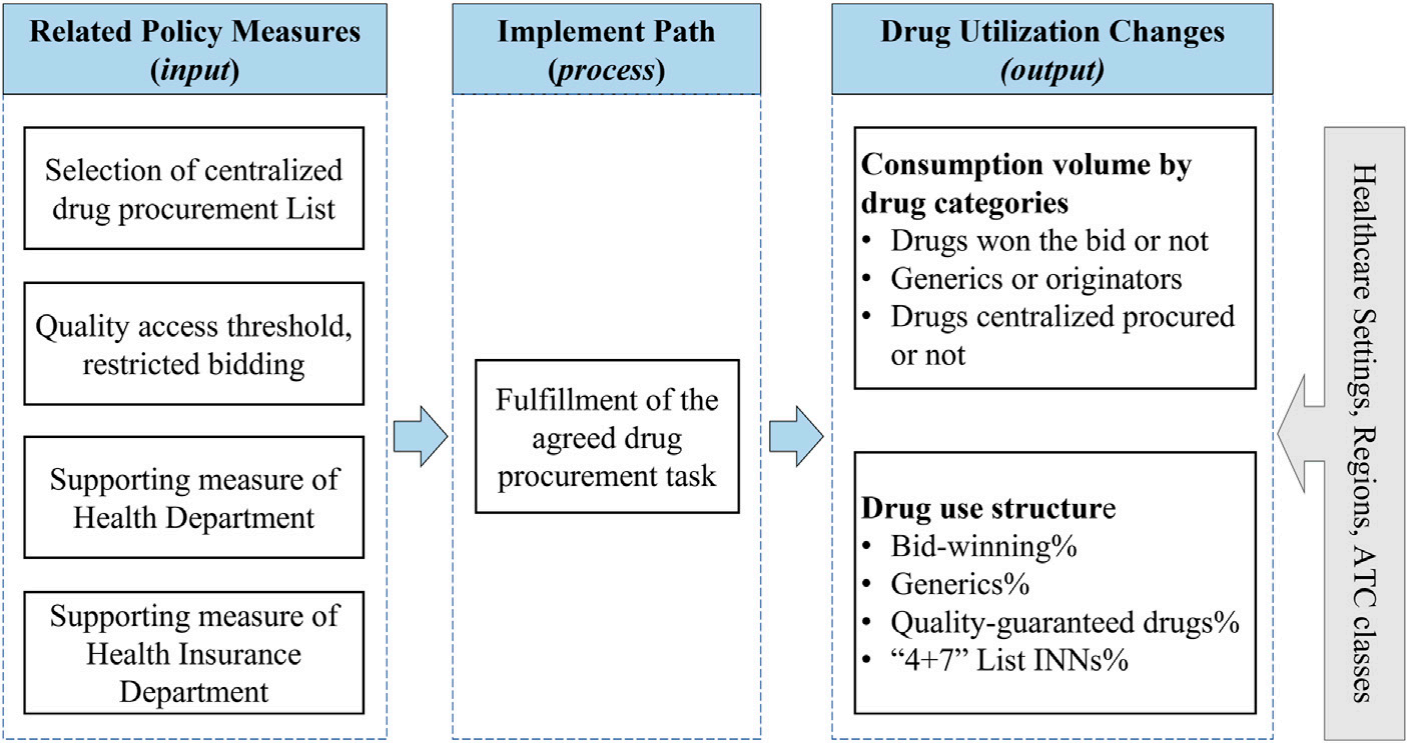
Research framework. Note: ATC, anatomical therapeutic chemical.

Metrics for measuring pharmaceutical policy outcomes linked to core objectives can be classified as a framework consisted of input, process, and output parameters. In this study, the abovementioned policy measures related to drug utilization were considered as input parameters. The process parameter refers to the path that leads to changes in drug use after the implementation of policy measures, defined as “medical institutions purchase and use contacted procurement quantity of the bid-winning drugs in the contracted procurement period.” Next, the output parameter refers to the outcomes emerged after the implementation of the policy over a specific period, which was defined as drug utilization changes. In this study, the drug utilization changes under policy implementation were measured from two dimensions: consumption volume dimension and drug use structure dimension, which were defined in detail below (the Methods section). Furthermore, we analyzed the change in drug use among different anatomical therapeutic chemical (ATC) classifications, different healthcare settings, and different geographical regions. Figure 1 outlined the framework of this study.

## Methods

### Study design

This study adopted a natural experimental study design with a standard difference-in-difference (DID) analysis method. One of the preconditions of constructing the standard DID model is that the target intervening measure only affects relevant factors in the treatment group and demonstrates no effect on the control group. Previous literature provided evidence that the influence of centralized procurement policy may involve different drug dimensions: drugs covered by the policy and those alternatives that were not, drugs that won the bid and those that did not, and generic and original drugs (Chen et al., 2020; Yang et al., 2021a; Wang et al., 2022). Therefore, in this study, the design of the DID model mainly discussed differences in regional and time dimensions. Further, we evaluated drug utilization changes in the treatment group (regions covered by the centralized procurement policy) vs. control group (regions where the policy did not cover) before and after policy implementation. The design of the empirical analysis strategy and the reporting of research results followed the reporting guideline for natural experiments issued by the Medical Research Council (Craig et al., 2012).

#### Intervention time point

In this study, the implementation of “4 + 7” bid-winning results was defined as the intervention measure. As the 11 “4 + 7” pilot cities start purchasing bid-winning drugs between 15 March 2019 and 1 April 2019, in this study, we determined March 2019 as the implementation ending time point of the “4 + 7” pilot.

#### Intervention group

In the present study, all eleven “4 + 7” pilot cities were assigned to the intervention group, namely Beijing, Shanghai, Tianjin, Chongqing, Guangzhou, Shenzhen, Xiamen, Shenyang, Dalian, Xi’an, and Chengdu. According to the geographical region of China, the 11 pilot cities are distributed in east China (Shanghai, Xiamen), North China (Beijing, Tianjin), Central China (Guangzhou, Shenzhen), Northeast China (Dalian, Shenyang), Southwest China (Chengdu, Chongqing), and Northwest China (Xi’an).

#### Control group

The determination of a comparable control group to the intervention group is the key step in natural experiment design. Considering China’s regional variation in drug use habits, pharmaceutical industry distribution, economic level, and health resources (Li et al., 2013; Zhang and Zhang, 2015; Yan and Yan, 2019), we first stratify the observation area samples by geographical regions, and then, we determine comparable control area samples within each geographical region. According to Li et al. (2021) and Tang (2016)’s method, the unweighted TOPSIS (technique for order performance by similarity to ideal solution) method was adopted to identify control samples with the highest matching degree (the closest TOPSIS score) among the provinces that did not implement “4 + 7” pilot as the control group. Nine matching variables were considered, including per capita gross domestic product (GDP), population size, number of health institutions, number of hospital beds, number of skilled health workers, number of licensed (assistant) doctors, per capital health expenditure, annual average clinical visits, and annual hospitalization rate. Twenty-one provinces that did not implement the “4 + 7” policy were initially available for matching, and finally, eleven provinces with the closest TOPSIS score of the pilot cities were included as the control group. Details of the TOPSIS results are listed in Supplementary Table S1.

### Data sources

The data used in this study came from the China Drug Supply Information Platform (CDSIP) (NHC, 2015), which covered the drug procurement order data of all provincial centralized procurement platforms across 31 provinces (autonomous regions and municipalities) in the mainland China. The data of CDSIP exhibit the features of great authenticity, integrality, and representativeness, and the details and sample coverage of the CDSIP database were introduced in the previous study by our team (Yang et al., 2022b).

The procurement data extracted from the CDSIP database include drug name, the name of medical institution, procurement date, dosage form, specification, packaging, manufacturer, unit price, procurement unit (by box, bottle, or branch), procurement quantity, procurement expenditures, etc. In the present study, we selected “4 + 7”-related drugs as study samples (Wang N. et al., 2021; Yang et al., 2022a; Wang et al., 2022), which were defined as drug INNs in the “4 + 7” procurement List (“4 + 7” List drugs) as well as their alternative drugs that have not yet been covered by the “4 + 7” procurement policy. Next, the identification of alternative drugs followed the definition of the NHSA in the Monitoring Plan for Centralized Drug Procurement and Use Pilot Work (NHSA, 2019b), which refers to the clinically substitutable drugs of the same kind with “4 + 7” List drugs. The list of included drugs is presented in Supplementary Table S2.

**FIGURE 2:**
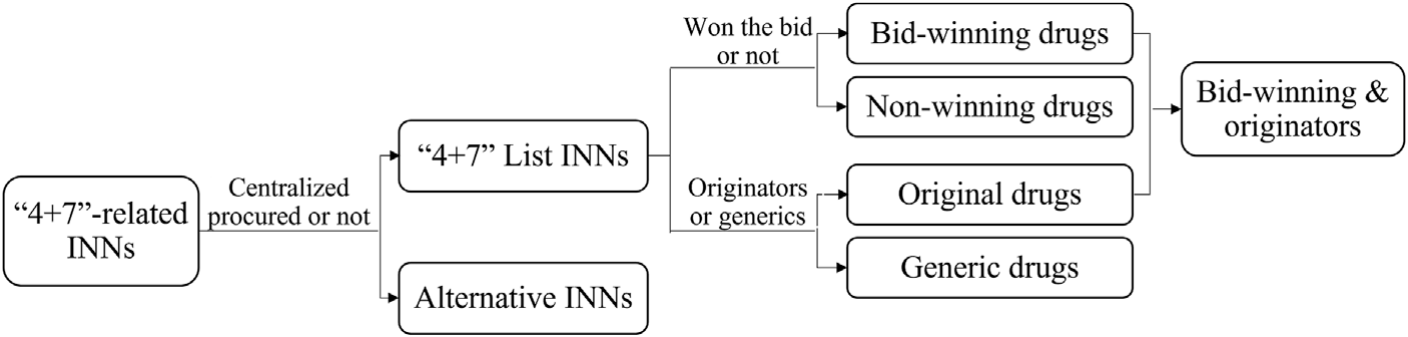
The classification of included drugs.

Then, the “4 + 7” List drugs were divided into bid-winning and non-winning drugs based on the “4 + 7” city procurement bid-winning results (Joint Procurement Office, 2018a), and they were sorted into off-patent original branded products and generic products according to the Catalogue of Marketed Drug in China (NMPA, 2017) (Figure 2). Since the Chinese government implemented the GCE work, generic drugs that pass the pharmacokinetics equivalence and bioequivalence trials are certified for quality and efficacy consistency to their corresponding originators. The assumption exists that certificated generics are of the same quality level as originators, and they demonstrate a higher quality level than uncertificated generics. Therefore, we defined bid-winning drugs and non-winning originators as relatively quality-guaranteed drugs, as only certificated generics and originators can participate and win the bid according to the policy requirements (General Office of the State Council, 2019a). In addition, included drug INNs were aggregated into 8 ATC groups: C-cardiovascular system (*n* = 8), N-nervous system (*n* = 7), L-antineoplastic and immunomodulating agents (*n* = 3), J-antiinfectives for systemic use (*n* = 3), A-alimentary tract and metabolism (*n* = 1), B-blood and blood forming organs (*n* = 1), M-musculoskeletal system (*n* = 1), and R-respiratory system (*n* = 1). Next, public medical institutions were divided into tertiary hospitals, secondary hospitals, and primary healthcare centers (PHCs). Then, finally, in this study, a total of 116 drug INNs (twenty-five “4 + 7” List drugs and 91 alternative drugs) were included.

### Outcome measures

The standardization of drug use quantity is the primary work of drug utilization research (Hollingworth and Kairuz, 2021). In this study, following the recommendation of the World Health Organization (WHO), we applied defined daily dose (DDD) (WHO Collaborating Centre for Drug Statistics Methodology, 2020) as the measurement unit to standardize the quantity of drugs used, to ensure the comparability of drugs in quantity with different generic names, dosage forms, and specifications. The DDD of several drugs, which could not be coded in WHO’s ATC/DDD Index 2021 system, was determined based on the recommended daily dosage in the manufacturers’ instructions, as approved by the China Food and Drug Administration (WHO Collaborating Centre for Drug Statistics Methodology, 2021). The calculation of drug use is as follows:

$$Y=\sum_{i=1}^{n}\left(\frac{U_{i}P_{i}}{{DDD}_{i}}\times N_{i}\right)$$ (1)

where *Y* is DDDs and represents the consumed volume of a certain drug (or a group of drugs); *DDD*_*i*_ refers to the DDD value of drug product *i*; $U_{i}$ refers to the unit ingredient of product *i*; $P_{i}$ refers to the packing specification of product *i*; and $N_{i}$ refers to the number of product *i*.

In addition to the primary volume indicator for drug use, four drug use structure indicators were included for measuring drug utilization by referring to government assessment documents (General Office of the State Council, 2019b; NHSA, 2019b) and relevant literature (Xie et al., 2021; Yang et al., 2022a; Luo et al., 2022; Wang et al., 2022), including the use proportion of bid-winning drugs, the proportion of generics, the proportion of bid-winning and originators, and the proportion of “4 + 7” List drugs. Also, an indicator—the “procurement completion rate”—was included as the *process* parameter according to the NHC documents (NHC, 2019a; NHC, 2019b).

$$\mathrm{procurement}\;\mathrm{completion}\;\mathrm{rate}=\frac{\mathrm{actual}\;\mathrm{procurement}\;\mathrm{volume}\;\mathrm{of}\;\mathrm{bid}-\mathrm{winning}\;\mathrm{drugs}}{\mathrm{agreed}\;\mathrm{procurement}\;\mathrm{volume}}\times 100\%$$ (2)

In Eq. 2, the “agreed procurement volume” refers to the purchase volume to be completed by the pilot cities as published by the Joint Procurement Office (2018b), which is generated based on reports from medical institutions of each pilot city. The “actual procurement volume of bid-winning drugs” refers to the volume of drugs actually purchased by each pilot city during the one-year procurement cycle.

$$\mathrm{bid}-\mathrm{winning}\;\mathrm{drugs}\%=\frac{\mathrm{volume}\;\mathrm{of}\;\mathrm{bid}-\mathrm{winning}\;\mathrm{drugs}}{\mathrm{volume}\;of^{\prime \prime }4+7^{\prime \prime }\;\mathrm{List}\;\mathrm{INNs}}\times 100\%$$ (3)

$$\mathrm{generics}\%=\frac{\mathrm{volume}\;\mathrm{of}\;\mathrm{generics}}{\mathrm{volume}\;of^{\prime \prime }4+7^{\prime \prime }\;\mathrm{List}\;\mathrm{INNs}}\times 100\%$$ (4)

$$\mathrm{bid}-\mathrm{winning}\&\mathrm{originators}\%=\frac{\mathrm{volume}\;\mathrm{of}\;\mathrm{bid}-\mathrm{winning}\&\mathrm{originators}}{\mathrm{volume}\;of^{\prime \prime }4+7^{\prime \prime }\;\mathrm{List}\;\mathrm{INNs}}\times 100\%$$ (5)

$$\prime \prime 4+7\prime \prime \;\mathrm{List}\;\mathrm{INNs}\%=\frac{\mathrm{volume}\;of^{\prime \prime }4+7^{\prime \prime }\;\mathrm{List}\;\mathrm{INNs}}{\mathrm{volume}\;of^{\prime \prime }4+7^{\prime \prime }\;\mathrm{List}\;\mathrm{and}\;\mathrm{alternative}\;\mathrm{INNs}}\times 100\%$$ (6)

In Eqs. 3–6, the “volume of “4 + 7” List INNs” refers to the volume of “4 + 7” List INNs purchased in a certain observation region in a certain time. The “volume of bid-winning drugs” refers to the volume of bid-winning drugs purchased in a certain observation region in a certain time. Next, the “volume of generics” refers to the volume of generic drugs in the “4 + 7” List purchased in a certain observation region at a certain time. The “volume of bid-winning and originators” refers to the overall volume of bid-winning drugs and non-winning original drugs in the “4 + 7” List purchased in a certain observation region at a certain time. Further, the “volume of “4 + 7” List and alternative INNs” refers to the overall volume of “4 + 7” List INNs and alternative INNs purchased in a certain observation region at a certain time, respectively.

### Statistical analysis

#### Descriptive analysis

First, we applied the descriptive statistical methods to quantify the change in drug use volume of each category and drug use structure in the pre- and post-intervention periods, as well as stratified changes by healthcare settings, geographical regions, and ATC classes. Next, to visualize the policy’s effects, we plotted monthly trends of drug use structure variables.

#### Difference-in-difference modeling

We adopted the DID approach to estimate the impact of the “4 + 7” pilot, where we performed generalized linear models to quantify the associations of policy intervention with the changes in the outcome indicators. The basic regression model is specified as follows:

$$Y_{\mathrm{it}}=\alpha +\beta \left(D_{i}\cdot T_{t}\right)+\mu_{\mathrm{it}}+\delta_{\mathrm{it}}+\varepsilon_{\mathrm{it}}$$ (7)

where *Y*_*it*_ refers to outcome variables of region *i* in month *t*. Next, $D_{i}$ is a dummy variable of policy intervention groups, coded 1 if region *i* belongs to the treatment group and coded 0 in the control group. $T_{t}$ is a dummy variable of policy intervention time, coded 0 in the month *i* before policy implementation (January 2018–February 2019) and coded 1 after policy intervention (March–December 2019). $\mu_{\mathrm{it}}$ and $\delta_{\mathrm{it}}$ are fixed effects of months and regions. $\varepsilon_{\mathrm{it}}$ refers to the random error term. $D_{i}\cdot T_{t}$ is the interaction term between study group and time, and its coefficient *β* refers to the DIDs effect associated with policy intervention.

#### Common pre-trend tests

Common trend refers to the idea that the treatment group would have evolved with the same trend as the control group with the absent of treatment, which is the premise of DID method to identify causal effects. Next, strictly, the common trend cannot be directly observed and tested, and it is usually done by common pre-trend tests to prove that the outcome variable demonstrates the same time-varying trend between the intervention group and control group in the pre-intervention period (Huang et al., 2022), with the following regression model:

$$Y_{\mathrm{it}}=\alpha +\sum_{s=1}^{T_{D}-2}\beta_{s}^{\mathrm{pre}}\left(D_{i}\cdot T_{t}^{s}\right)+\sum_{\mathrm{s=}T_{D}}^{T}\beta_{s}^{\mathrm{post}}\left(D_{i}\cdot T_{t}^{s}\right)+\theta W_{\mathrm{it}}+\mu_{\mathrm{it}}+\delta_{\mathrm{it}}+\varepsilon_{\mathrm{it}}$$ (8)

where $Y_{\mathrm{it}}$ refers to outcome variables. $D_{i}$ is a dummy variable of policy intervention groups, with the intervention group (“4 + 7” pilot cities) being coded as 1 and the control group coded as 0. $T_{t}^{s}$ is the time dummy of period *s*. $\beta_{s}^{\mathrm{pre}}$ and $\beta_{s}^{\mathrm{post}}$ refer to the differences of outcome variables between the intervention group and the control group before and after policy implementation in period *s*, compared with the differences of outcome variables in the base period (assigned to the first month of observation period). If the coefficient $\beta_{s}^{\mathrm{pre}}$ demonstrates no statistical significance, it indicates a common trend of the corresponding outcome variable in intervention and control group.

## Results

### Completion rate of agreed procurement quantity

**FIGURE 3:**
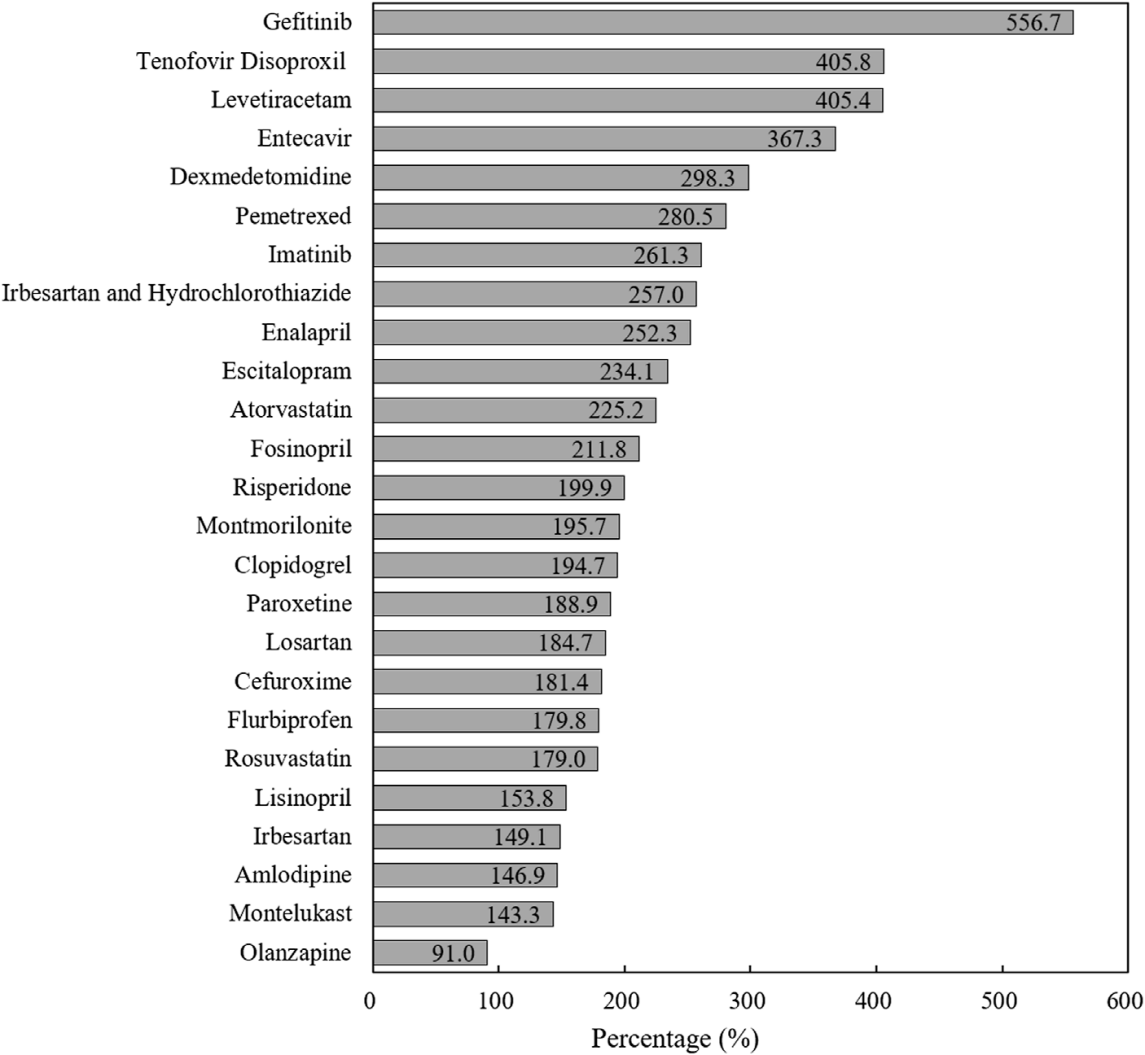
The completion rate of agreed procurement quantity of each “4 + 7” List drug during one-year agreement period.

We calculated the completion rate of pooled procured drugs based on the annual agreed procurement volume of each drug (by INN) in each pilot city (Joint Procurement Office, 2018b). During one-year policy implementation (April 2019–March 2020), the actual procurement volume of bid-winning drugs reached 2.37 billion DDDs in pilot cities, with a total procurement completion rate of 191.4%. Figure 3 indicates the completion rate of each “4 + 7” bid-winning drug. Except for Olanzapine (91.0%), the procurement completion rates of other 24 drugs exceeded 100%, ranging from 143.3 to 556.7%. Next, a completion rate of more than 200% was observed in twelve drugs (12/25). Also, the separated analysis of each pilot city indicated that all 11 pilot cities fulfilled their agreed procurement tasks in excess.

### Drug utilization changes

#### Bid-winning and non-winning drugs

**FIGURE 4:**
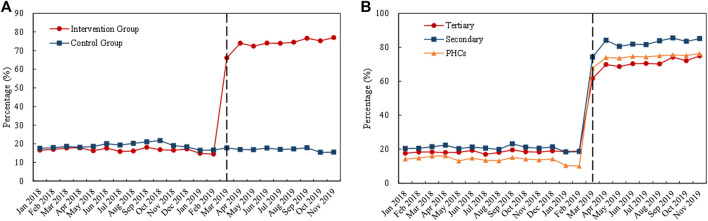
Trends in the volume proportion of bid-winning drugs during January 2018 to November 2019: **(A)** volume proportion of bid-winning drugs in the intervention group and control group **(B)** volume proportion of bid-winning drugs in intervention group by healthcare setting. *Note*: PHCs, primary healthcare centers.

Figure 4 visualizes the trends in monthly volume proportion of bid-winning drugs. Before policy intervention, the proportion in the intervention group and control group generally remained the same level (about 20%). After policy intervention in March 2019, the proportion in the intervention group drastically increased to about 70%, while the proportion in the control group maintained at previous level (Figure 4A). Under policy intervention, in all three types of medical institutions, markable increases were found in the volume proportion of bid-winning drugs (Figure 4B).

As shown in Table 1, DID analysis revealed that the monthly volume of bid-winning drugs increased significantly (*β* = 1.62, *p* < 0.001) after policy intervention, with a 405.31% increment when some transformations of the coefficients were made (Kim and Skordis-Worrall, 2017; Zhang et al., 2017; Li et al., 2021). The volume of non-winning drugs was associated with a 62.28% reduction (*β* = −0.98, *p* < 0.001). The volume proportion of bid-winning drugs raised from 17.03% in the pre-intervention period to 73.61% in the post-intervention period, and the increase was detected with significance in the DID analysis (*β* = 1.48, *p* < 0.001).

**TABLE 1:** Impact of the “4 + 7” pilot on monthly volumes of bid-winning or non-winning drugs, stratified by healthcare settings.

Categories	Descriptive change (million DDDs)			DID estimate		
	Pre	Post	Change (%)	β	95% *CI*	Change (%)
Overall						
Bid-winning drugs	345.00	1690.00	389.86	1.62	(1.45, 1.79)^***^	405.31
Non-winning drugs	1681.00	606.00	−63.95	−0.98	(−1.21, −0.74)^***^	−62.28
Bid-winning%	17.03	73.61	56.58	1.48	(1.39, 1.57)^***^	337.54
Bid-winning drugs						
Tertiary hospitals	150.30	675.50	349.43	1.50	(1.32, 1.68)^***^	346.83
Secondary hospitals	64.34	297.40	362.20	1.02	(0.78, 1.25)^***^	175.94
PHCs	130.40	717.30	450.08	2.29	(1.99, 2.60)^***^	890.46
Non-winning drugs						
Tertiary hospitals	668.80	287.90	−56.95	−0.77	(−0.99, −0.55)^***^	−53.79
Secondary hospitals	239.10	64.90	−72.86	−1.97	(−2.23, −1.70)^***^	−86.00
PHCs	773.50	253.20	−67.27	−1.04	(−1.40, -0.68)^***^	−64.62
Bid-winning%						
Tertiary hospitals	18.35	70.12	51.76	1.29	(1.20, 1.39)^***^	264.01
Secondary hospitals	21.21	82.09	60.88	1.52	(1.41, 1.63)^***^	355.85
PHCs	14.43	73.91	59.48	1.83	(1.65, 2.01)^***^	523.39

Note: ^*^ *p*< 0.05.

^**^ *p*< 0.01.

^***^ *p*< 0.001.

Pre refers to March–November 2018; Post refers to March–November 2019; Bid-winning% refers to the volume proportion of bid-winning drugs in the “4 + 7” List drugs.

DDDs, defined daily doses; DID, difference-in-difference; CI, confidence interval; PHCs, primary healthcare lefts.

Considering the type of medical institution, significant increases in bid-winning drugs were observed in the healthcare setting of tertiary hospitals (346.83%), secondary hospitals (175.94%), and PHCs (890.46%) (all *p*-values < 0.001). Significant decreases of non-winning drugs were observed in tertiary hospitals (−53.79%), secondary hospitals (−86.00%), and PHCs (−64.62%) (all *p*-values < 0.001). As for the volume proportion of bid-winning drugs, a prominent increase of 264.01, 355.85, and 523.39% were detected in tertiary hospitals (*β* = 1.29, *p* < 0.001), secondary hospitals (*β* = 1.52, *p* < 0.001), and PHCs (*β* = 1.83, *p* < 0.001), respectively.

#### Generic and original drugs

**FIGURE 5:**
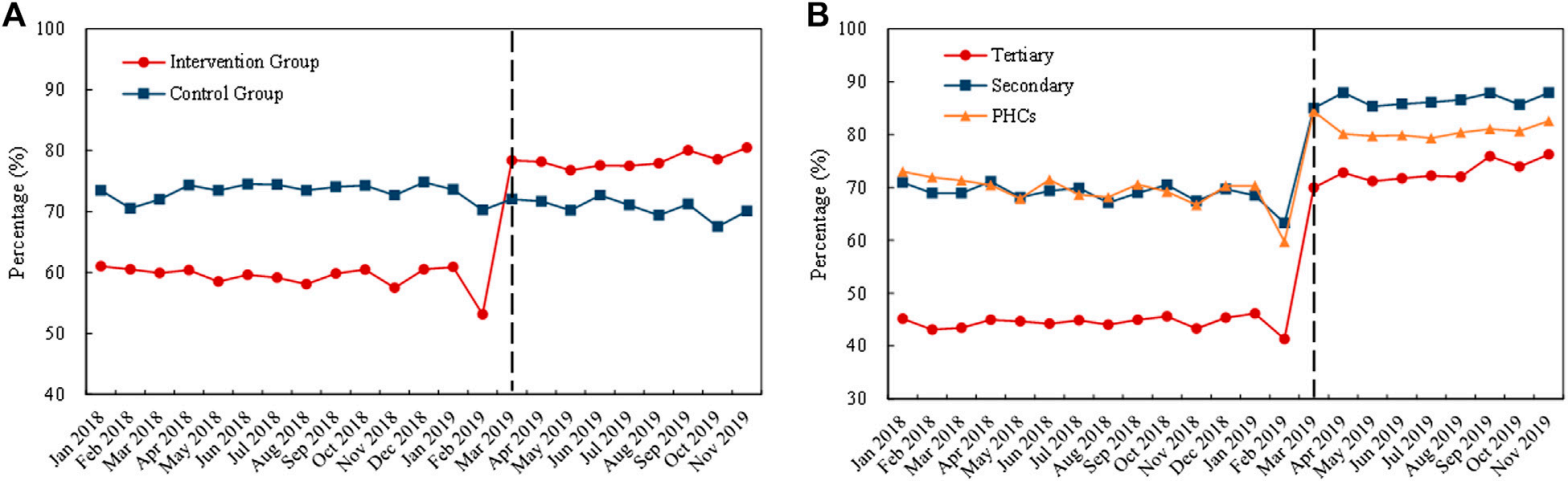
Trends in the volume proportion of generic drugs during January 2018 to November 2019: **(A)** volume proportion of generic drugs in the intervention group and control group **(B)** volume proportion of generic drugs in intervention group by healthcare setting. Note: PHCs, primary healthcare centers.

Figure 5 outlines the monthly trends of volume proportion of generic drugs. During the whole observation period, the proportion in the control group remained stable, while the proportion in the intervention group increased suddenly after the implementation of the policy in March 2019 (Figure 5A). Among the medical institutions, the proportion of generic drugs was lower in tertiary hospitals than in secondary hospitals and PHCs, and the proportion increased remarkedly in all healthcare settings with the implementation of the “4 + 7” pilot (Figure 5B).

Table 2 demonstrates the changing pattern in generic and original drugs. After policy intervention, the procurement volume of generic drugs in the “4 + 7” List INN increased by 67.53% (*β* = 0.52, *p* < 0.001), while original drugs decreased by 26.88% (*β* = −0.31, *p* = 0.006). Next, the volume proportion of generic drugs in the “4 + 7” List INN increased from 59.23% in the pre-intervention period to 78.44% in the post-intervention period, and the increment was statistically significant (*β* = 0.24, *p* < 0.001).

**TABLE 2:** Impact of the “4 + 7” pilot on monthly volumes of generic or original drugs, stratified by healthcare settings.

Categories	Descriptive change (million DDDs)				DID estimate	
	Pre	Post	Change (%)	*β*	95% *CI*	Change (%)
Overall						
Generics	1200.00	1801.00	50.08	0.52	(0.34, 0.69)^∗∗∗^	67.53
Originators	826.80	495.00	−40.13	−0.31	(−0.54, −0.09)^∗∗^	−26.88
Generics%	59.23	78.44	19.21	0.24	(0.20, 0.28)^∗∗∗^	27.12
Generics						
Tertiary hospitals	363.90	701.90	92.88	0.61	(0.42, 0.79)^∗∗∗^	83.68
Secondary hospitals	209.30	313.30	49.69	−0.12	(−0.35, 0.11)	−11.57
PHCs	626.60	786.00	25.44	0.60	(0.34, 0.86)^∗∗∗^	81.85
Originators						
Tertiary hospitals	455.10	261.50	−42.54	−0.24	(−0.45, −0.04)^∗^	−21.65
Secondary hospitals	94.13	48.98	−47.97	−1.17	(−1.43, 0.92)^∗∗∗^	−69.06
PHCs	277.30	184.50	−33.47	−0.03	(−0.39, 0.34)	−2.57
Generics%						
Tertiary hospitals	44.43	72.86	28.42	0.31	(0.27, 0.34)^∗∗∗^	35.66
Secondary hospitals	68.98	86.48	17.49	0.23	(0.17, 0.29)^∗∗∗^	25.86
PHCs	69.32	80.99	11.67	0.11	(0.07, 0.16)^∗∗∗^	12.08

∗ Note: *p* < 0.05.

∗∗ *p* < 0.01.

∗∗∗ *p* < 0.001.

Pre refers to March–November 2018; Post refers to March–November 2019; Generics% refers to the volume proportion of generic drugs in the “4 + 7” List drugs.

DDDs, defined daily doses; DID, difference-in-difference; *CI*, confidence interval; PHCs, primary healthcare lefts.

Significant increases of 83.68% and 81.85% were associated with the volume of generic drugs in tertiary hospitals (*β* = 0.61, *p* < 0.001) and PHCs (*β* = 0.60, *p* < 0.001), respectively, while the change in secondary hospitals was not statistically significant (*β* = −0.12, *p* = 0.296). The volume of original drugs significantly decreased by 21.65% and 69.06% in tertiary (*β* = −0.24, *p* = 0.022) and secondary (*β* = −1.17, *p* < 0.001) hospitals, respectively, while the volume in PHCs exhibited no significant change (*β* = −0.03, *p* = 0.890). The volume proportion of generic drugs increased by 28.42 (tertiary hospitals), 17.49 (secondary hospitals), and 11.67 (PHCs) percentage points, and the increases were significant from the DID analysis (all *p*-values < 0.001).

#### Use proportion of bid-winning and original drugs

**FIGURE 6:**
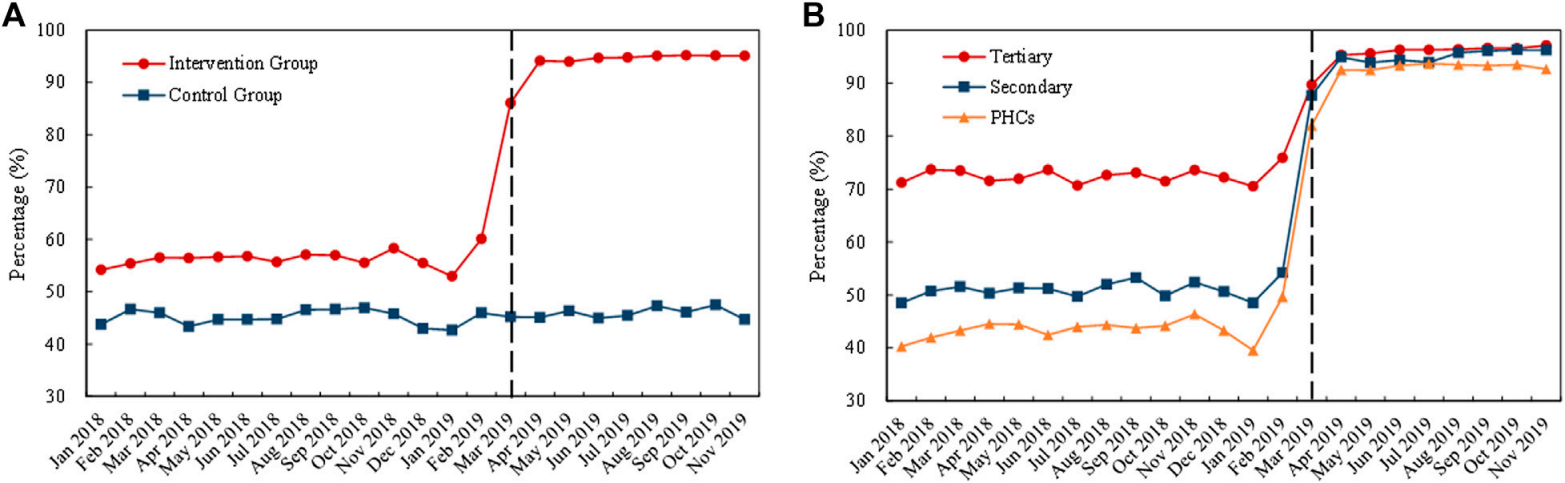
Trends in the volume proportion of bid-winning and originators during January 2018 to November 2019: **(A)** volume proportion of bid-winning and originators in the intervention group and control group **(B)** volume proportion of bid-winning and original drugs in intervention group by healthcare setting. Note: PHCs, primary healthcare centers.

Figure 6 displays the monthly trends of volume proportion of bid-winning and original drugs. The proportion in the control group remained stable (about 45%) during the whole observation period. The proportion in the intervention group increased remarkedly with the implementation of the “4 + 7” pilot in March 2019 (Figure 6A). Among the medical institutions, before policy intervention, the highest proportion in the intervention group was observed in tertiary hospitals (about 70%), followed by secondary hospitals (about 50%) and PHCs (about 40%). With the implementation of the “4 + 7” pilot in March 2019, the proportion in all types of medical institutions prominently increased, up to approximately 95% (Figure 6B).

As shown in Table 3, the volume proportion of bid-winning and original drugs in the “4 + 7” List INN increased from 56.69% in the pre-intervention period to 93.61% in the post-intervention period, with a significant increase of 83.31% (*β* = 0.61, *p* < 0.001). Further, an increase in bid-winning and original drugs’ volume proportions were demonstrated in all types of medical institutions, and the figure reached 95.42% (tertiary hospitals), 94.13% (secondary hospitals), and 91.63% (PHCs) in the post-intervention period. Next, DID analysis revealed that the proportion significantly increased by 53.88%, 89.27%, and 206.18% in tertiary hospitals (*β* = 0.43, *p* < 0.001), secondary hospitals (*β* = 0.64, *p* < 0.001), and PHCs (*β* = 1.12, *p* < 0.001), respectively.

**TABLE 3:** Impact of the “4 + 7” pilot on the volume proportion of bid-winning and originators, stratified by healthcare settings.

Categories	Descriptive change (million DDDs)			DID estimate		
	Pre	Post	Change (%)	*β*	95% *CI*	Change (%)
Overall	56.69	93.61	36.93	0.61	(0.57, 0.64)^***^	83.31
Tertiary hospitals	72.49	95.42	22.93	0.43	(0.41, 0.46)^***^	53.88
Secondary hospitals	51.32	94.13	42.82	0.64	(0.59, 0.69)^***^	89.27
PHCs	44.19	91.63	47.45	1.12	(0.97, 1.27)^***^	206.18

Note: * *p* < 0.05.

**p < 0.01.

***p < 0.001.

Pre refers to March–November 2018; Post refers to March–November 2019.

DID, difference-in-difference; PHCs, primary healthcare lefts; *CI*, confidence interval.

#### “4 + 7” List and alternative drugs

**FIGURE 7:**
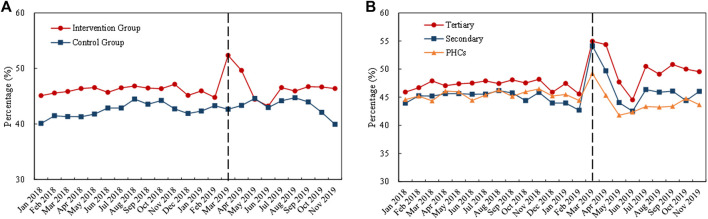
Trends in the volume proportion of “4 + 7” List drugs during January 2018 to November 2019: **(A)** volume proportion of “4 + 7” List drugs in the intervention group and control group **(B)** volume proportion of “4 + 7” List drugs in intervention group by healthcare setting. Note: PHCs, primary healthcare centers.

Figure 7 demonstrates the monthly trends of volume proportion of “4 + 7” List drugs. The proportion in the intervention group prominently improved in the early periods of policy implementation (March to April 2019) (Figure 7A). Also, a similar increment was observed in three medical institution types in this period (Figure 7B).

Table 4 presents the change of “4 + 7” List drugs and their alternative drugs. After policy intervention, the volume of “4 + 7” List drugs significantly increased by 34.45% (*β* = 0.30, *p* = 0.002). The volume change of alternative drugs was not statistically significant (*β* = 0.01, *p* = 0.956). As for the proportion of “4 + 7” List drugs in the total volume of “4 + 7” List drugs and alternative drugs, a prominent increase was observed after intervention (*β* = 0.18, *p* < 0.001).

**TABLE 4:** Impact of the “4 + 7” pilot on monthly volumes of “4 + 7” List drugs and their alternative drugs, stratified by healthcare settings.

Categories	Descriptive change (million DDDs)			DID estimate		
	Pre	Post	Change (%)	*β*	95% *CI*	Change (%)
Overall						
“4 + 7” List INNs	2026.00	2296.00	13.33	0.30	(0.11, 0.48)^**^	34.45
Alternative INNs	2340.00	2591.00	10.73	0.01	(−0.16, 0.17)	0.50
“4 + 7” List INNs%	46.40	46.98	0.58	0.18	(0.15, 0.22)^***^	20.08
“4 + 7” List INNs						
Tertiary hospitals	819.00	963.40	17.63	0.32	(0.14, 0.51)^**^	37.99
Secondary hospitals	303.40	362.30	19.41	−0.36	(−0.59, −0.13)^**^	−30.44
PHCs	903.90	970.50	7.37	0.49	(0.23, 0.75)^***^	62.74
Alternative INNs						
Tertiary hospitals	898.00	952.60	6.08	0.14	(−0.04, 0.31)	14.57
Secondary hospitals	362.90	412.40	13.64	−0.58	(−0.79, −0.38)^***^	−44.23
PHCs	1079.00	1226.00	13.62	−0.02	(−0.27, 0.24)	−1.69
“4 + 7” List INNs%						
Tertiary hospitals	47.70	50.28	2.58	0.11	(0.08, 0.14)^***^	11.52
Secondary hospitals	45.53	46.77	1.24	0.18	(0.13, 0.22)^***^	19.12
PHCs	45.58	44.17	−1.41	0.40	(0.33, 0.47)^***^	48.59

Note: * *p* < 0.05.

**p < 0.01.

***p <0.001.

Pre refers to March–November 2018; Pos^a^efers to March–November 2019; “4 + 7” List INNs% refers to the volume proportion of “4 + 7” List INNs, in the total volume of “4 + 7” List and alternative INNs.

DDDs, defined daily doses; DID, difference-in-difference; *CI*, confidence interval; INN, international nonproprietary name; PHCs, primary healthcare lefts.

In different healthcare settings, the volume of “4 + 7” List drugs significantly increased by 37.99% in tertiary hospitals (*β* = 0.32, *p* = 0.001) and 62.74% in PHCs (*β* = 0.49, *p* < 0.001), and it significantly decreased by 30.44% (*β* = −0.36, *p* = 0.002) in secondary hospitals after policy intervention. The volume of alternative drugs significantly decreased by 44.23% in secondary hospitals (*β* = −0.58, *p* < 0.001), and no significant changes were seen in tertiary hospitals (*β* = 0.14, *p* = 0.128) and PHCs (*β* = −0.02, *p* = 0.895). The volume proportion of “4 + 7” List drugs increased by 11.52%, 19.12%, and 48.59% in tertiary hospitals (*β* = 0.11, *p* < 0.001), secondary hospitals (*β* = 0.18, *p* < 0.001), and PHCs (*β* = 0.40, *p* < 0.001), respectively.

### Drug utilization changes by subgroup

#### Geographical region

Table 5 demonstrates the change in drug utilization among different geographical regions. After policy intervention, the volume proportion of bid-winning drugs significantly increased in all six regions: 496.55% (east China), 269.14% (North China), 272.48% (Central China), 311.65% (Northeast China), 404.30% (Southwest China), and 330.60% (Northwest China) (all *p*-values < 0.001). In the post-intervention period, bid-winning drugs’ volume proportion reached between 62.20% and 84.94% in the six regions.

**TABLE 5:** Subgroup analyses on the impacts of “4 + 7” pilot on drug use structure by geographical region.

Regions	Bid-winning%			Generics%			Bid-winning & originators%			“4 + 7” list INNs%		
	Pre	Post	Change (%)	Pre	Post	Change (%)	Pre	Post	Change (%)	Pre	Post	Change (%)
**Descriptive change**												
East China	18.45	84.94	66.49	76.20	88.89	12.68	41.52	95.05	53.53	45.21	40.77	-4.44
North China	17.15	62.20	45.05	50.05	68.73	18.67	65.38	91.49	26.12	45.91	46.55	0.64
Central China	8.48	77.78	69.30	46.53	78.58	32.05	61.24	97.68	36.44	52.79	55.87	3.08
Northeast China	14.61	78.64	64.03	53.51	82.11	28.60	60.05	94.79	34.74	39.31	46.18	6.87
Southwest China	21.74	81.33	59.60	66.25	87.10	20.85	55.06	92.95	37.89	48.23	52.48	4.25
Northwest China	33.25	83.43	50.18	63.87	85.11	21.24	67.90	97.47	29.57	38.78	53.48	14.69
** Regions**	**Bid-winning%**			**Generics%**			**Bid-winning and Originators%**			**“4 + 7” List INNs%**		
	***β***	***p***	**Change (%)**	***β***	***p***	**Change (%)**	***β***	***p***	**Change (%)**	***β***	***p***	**Change (%)**
**DID estimate**												
East China	1.79	**< 0.001**	496.55	0.21	**< 0.001**	22.88	0.82	**< 0.001**	126.82	0.03	0.572	2.74
North China	1.31	**< 0.001**	269.14	0.21	**< 0.001**	23.37	0.48	**< 0.001**	62.09	0.18	**< 0.001**	19.60
Central China	1.32	**< 0.001**	272.48	-0.001	0.979	-0.10	0.86	**< 0.001**	136.08	0.36	**< 0.001**	42.62
Northeast China	1.42	**< 0.001**	311.65	0.45	**< 0.001**	57.46	0.37	**< 0.001**	44.63	0.09	**0.002**	8.98
Southwest China	1.62	**< 0.001**	404.30	0.31	**< 0.001**	36.21	0.59	**< 0.001**	79.68	0.20	**< 0.001**	22.63
Northwest China	1.46	**< 0.001**	330.60	0.34	**< 0.001**	40.21	0.50	**< 0.001**	64.38	0.29	**< 0.001**	33.24

Note: Bold values indicate regression coefficients with statistical significance (*p*-value < 0.05). Pre refers to March–November 2018; Post refers to March–November 2019; Bid-winning% refers to the volume proportion of bid-winning drugs in the “4 + 7” List INNs; Generics% refers to the volume proportion of generic drugs in the “4 + 7” List INNs; Bid-winning & Originators% refers to the volume proportion of bid-winning products or original products in the “4 + 7” List INNs; “4 + 7” List INNs% refers to the volume proportion of “4 + 7” List INNs, in the total volume of “4 + 7” List and alternative INNs.

INN, international nonproprietary name; DID, difference-in-difference.

Among the six geographical regions, the change in volume proportion of generic drugs in central China was not statistically significant (*β* = −0.001, *p* = 0.979), while significant increases were observed in other five regions (all *p*-values < 0.001): 22.88% (east China), 23.37% (north China), 57.46% (northeast China), 36.21% (southwest China), and 40.21% (northwest China).

The volume proportion of bid-winning and original drugs significantly increased in all the six regions after policy intervention: 126.82% (east China), 62.09% (north China), 136.08% (central China), 44.63% (northeast China), 79.68% (southwest China), and 64.38% (northwest China) (all *p*-values < 0.001). Next, in the post-intervention period, the volume proportion of bid-winning and original drugs ranged from 91.49% to 97.68% among the six regions.

Among the six geographical regions, the change in volume proportion of “4 + 7” List drugs in east China was not statistically significant (*β* = 0.03, *p* = 0.572), while prominent increases were detected in other five regions (all *p*-values < 0.01): 19.60% (north China), 42.62% (central China), 8.98% (northeast China), 22.63% (southwest China), and 33.24% (northwest China).

#### ATC classification

In this study, the included drugs covered eight ATC classifications. As shown in Table 6, the volume proportion of bid-winning drugs significantly increased after policy intervention in all the ATC classes (all *p*-values < 0.001).

**TABLE 6:** Subgroup analyses on the impacts of “4 + 7” pilot on drug use structure by ATC classification.

ATC class	Bid-winning%			Generics%			Bid-winning & originators%			“4 + 7” list INNs%		
	Pre	Post	Change (%)	Pre	Post	Change (%)	Pre	Post	Change (%)	Pre	Post	Change (%)
**Descriptive change**												
ATC-C	13.21	72.65	59.44	57.90	77.35	19.45	54.32	93.83	39.51	49.35	48.52	−0.84
ATC-N	31.84	67.08	35.24	72.18	82.50	10.31	59.65	84.58	24.93	33.67	33.44	−0.23
ATC-L	25.96	65.91	39.96	72.43	59.17	−13.26	34.89	73.02	38.14	73.00	69.90	−3.10
ATC-J	28.32	84.57	56.25	78.00	89.85	11.85	50.32	94.70	44.39	53.58	68.23	14.64
ATC-A	2.68	73.46	70.78	71.15	93.01	21.87	31.53	80.44	48.91	19.40	17.42	−1.98
ATC-B	36.83	75.42	38.60	58.77	78.54	19.77	78.08	96.90	18.82	34.59	38.87	4.28
ATC-M	97.86	93.92	−3.94	2.14	6.08	3.94	97.86	93.92	−3.94	19.92	18.78	−1.14
ATC-R	0.00	69.99	69.99	39.43	76.73	37.29	60.57	93.26	32.69	78.28	73.25	−5.03

**ATC class**	**Bid-winning%**			**Generics%**			**Bid-winning and Originators%**			**“4 + 7” List INNs%**		
	***β***	***p***	**Change (%)**	***β***	***p***	**Change (%)**	***β***	***p***	**Change (%)**	***β***	***p***	**Change (%)**
**DID estimate**												
ATC-C	1.69	**< 0.001**	444.12	0.26	**< 0.001**	29.43	0.66	**< 0.001**	92.90	0.14	**< 0.001**	15.03
ATC-N	0.72	**< 0.001**	104.83	0.01	0.541	0.90	0.57	**< 0.001**	76.12	0.09	**0.003**	9.53
ATC-L	0.93	**< 0.001**	153.96	−0.38	**< 0.001**	−31.89	0.88	**< 0.001**	141.09	−0.06	**0.002**	-5.73
ATC-J	0.87	**< 0.001**	137.98	0.03	**0.004**	3.46	0.77	**< 0.001**	116.84	0.11	**< 0.001**	11.74
ATC-A	2.48	**< 0.001**	1090.55	0.36	**< 0.001**	43.19	1.02	**< 0.001**	176.77	−0.23	**< 0.001**	−20.55
ATC-B	1.05	**< 0.001**	186.91	0.67	**< 0.001**	96.21	0.16	**< 0.001**	17.47	0.50	**< 0.001**	65.37
ATC-M	0.12	**< 0.001**	13.20	−0.39	0.249	−32.50	0.24	**< 0.001**	26.87	0.71	**< 0.001**	103.60
ATC-R	4.60	**< 0.001**	9858.38	0.65	**< 0.001**	91.17	0.47	**< 0.001**	59.84	−0.01	0.709	−1.19

Note: Bold values indicate regression coefficients with statistical significance (*p*-value < 0.05). Pre refers to March–November 2018; Post refers to March–November 2019; Bid-winning% refers to the volume proportion of bid-winning drugs in the “4 + 7” List INNs; Generics% refers to the volume proportion of generic drugs in the “4 + 7” List INNs; Bid-winning & Originators% refers to the volume proportion of bid-winning products or original products in the “4 + 7” List INNs; “4 + 7” List INNs% refers to the volume proportion of “4 + 7” List INNs, in the total volume of “4 + 7” List and alternative INNs.

ATC, anatomical therapeutic chemical; INN, international nonproprietary name; DID, difference-in-difference.

As for the volume proportion of generic drugs, no significant change was observed in ATC-N (*β* = 0.01, *p* = 0.541) and ATC-M (*β* = -0.39, *p* = 0.249). By contrast, significant increases were found in ATC-C (29.43%), ATC-J (3.46%), ATC-A (43.19%), ATC-B (96.21%), and ATC-R (91.17%), while a significant decrease was found in ATC-L (-31.89%).

For the volume proportion of bid-winning and original drugs, prominent increases were detected in all eight ATC classes (all *p*-values < 0.001), with the increment ranged from 17.47% to 176.77%. During the post-intervention period, the proportion of bid-winning and original drugs in eight ATC classes was between 73.02% and 96.90%.

In terms of the volume proportion of “4 + 7” List drugs, among the eight ATC classes, five (ATC-C, ATC-N, ATC-J, ATC-B, and ATC-M) demonstrated significant increases (all *p*-values < 0.05), two (ATC-L and ATC-A) showed significant decreases (all *p*-values < 0.05), and one (ATC-R) indicated no significant change (*β* = −0.01, *p* = 0.709).

### Common pre-trends tests for DID

According to the direct observation of the monthly trend chart above, it can be shown that, to some extent, the monthly trends of change in drug use were similar between the intervention group and control group prior to the “4 + 7” pilot implementation. Furthermore, we conducted common pre-trends tests for each outcome variable based on Eq. 8. As shown in Supplementary Figure S1, the coefficients of the interaction terms were all statistically insignificant in the pre-intervention periods—the point estimates and 95% confidence intervals of the interaction terms’ coefficients were not different from zero. Next, the results appeared that the outcome variables demonstrate the same time-varying trend between the intervention group and control group before policy implementation, thus clearly suggesting that the common trends assumption could not be rejected.

## Discussion

In this study, we quantified the change in drug utilization in China’s public medical institutions under the impact of the “4 + 7” centralized procurement policy, by using data of drug procurement order from an authoritative national database. Natural experimental design and difference-in-difference method were applied to estimate policy impacts. Overall, within the one-year agreed procurement period, the procurement tasks of bid-winning drugs were well completed in each “4 + 7” pilot city. After the implementation of the “4 + 7” policy, the use of policy-related drugs in China’s public medical institutions significantly changed, where drug use became more concentrated on bid-winning drugs, generic drugs, quality-guaranteed drugs, and drug INNs covered by centralized procurement list. Besides, a gradually decreasing difference existed in drug use structure among different healthcare settings and geographical areas.

First of all, results of this study showed that the accumulative actual procurement volume of bid-winning drugs in “4 + 7” pilot cities during the one-year procurement period (April 2019–March 2020) reached about two times the agreed volume; the procurement of a majority of drug INNs were also over-fulfilled. The present findings are generally consistent with the findings reported by NHSA (2020), reflecting good policy acceptance and compliance of healthcare providers. However, our investigation revealed that the published agreed procurement volume in the 11 pilot cities only accounted for approximately 45% of the actual procurement volume in 2018 (Joint Procurement Office, 2018b), which is markedly lower than the projected amount of 60–70% (General Office of the State Council, 2019a), and the underreporting was particularly prominent in several pilot cities. This finding may implicate the phenomenon of underreporting of procurement demands in public medical institutions to relieve their pressure of assessment, which was also reported in a previous study (Yu, 2020). Next, the accurate identification of demands for drugs is the foundation for conducting centralized drug bidding and procurement, as well as for assessing the medical institutions. Therefore, in the future, to promote authentic reporting of drug use demands in medical institutions, a more comprehensive mechanism for reporting drug procurement volume and a reform of assessment approach are warranted.

Second, a significant increase was found in usage of bid-winning drugs following the policy intervention, while the opposite trend was observed in the non-winning drugs. As a consequence, bid-winning drugs became more dominant in use among the centralized procurement drugs, increasing from 14 to 74%. Also, these results are in line with the body of literature (Chen et al., 2020; Yang et al., 2021a; Chen et al., 2021; Wen et al., 2021; Yang et al., 2022a; Wang et al., 2022). In particular, such changes were found to be more prominent in PHCs, compared to those in secondary and tertiary hospitals, which indicated that the bid-winning drugs among the centralized procurement drugs may be more reflective to the drug demands at PHC level. Following the implementation of the “4 + 7” policy, the bid-winning drugs have become more accessible at community level (NHSA, 2022), which complies with the original policy intention of improving drug accessibility.

Third, we also observed a significant increase in usage of generic drugs under the impact of the “4 + 7” policy implementation, while the opposite trend was seen in the original drugs. The usage of generic drugs has become more dominant, increasing from 59% to 78%; the increment was the largest in tertiary hospitals, followed in order by secondary hospitals and PHCs. In China, the high reliance of use in original drugs has long existed, especially in large hospitals; also, an increasing use of original drugs is found year by year (Li et al., 2013; Zeng, 2013; Tang, 2016; Li et al., 2021; Huang et al., 2022), which appears to be contradicted to the situation in the United State where the generic drugs reached 90% of the prescriptions (FDA, 2019). Next, the low utilization rate of the generic drugs in China could be attributed to a number of factors, including the lack of sufficient understanding in the trust to the quality and efficacy of domestic generic drugs among the physicians, pharmacists, and patients (Oncu et al., 2020; Li et al., 2021), as well as the incentives for markups or rebates of high-priced drugs (such as the imported original drugs) (Zeng et al., 2014). After the implementation of the “4 + 7” centralized procurement policy, the long-standing problem of low utilization rate of generic drugs in public hospitals has been reversed; also, the variations in the utilization rate of generic drugs among healthcare settings and geographical regions also gradually reduced. In the future, persistent publicity and education of knowledge on generic drugs are needed to further reverse the misunderstandings of the generic drugs among the general public (Qu et al., 2021). Moreover, further improving the establishment of drug quality standard is urgent, to promote the monitoring and evaluation on the efficacy and safety of generic drugs that passed the GCE using real-world data and to consolidate the foundation for the substitution use of generic drugs.

Forth, since the centrally procured bid-winning drugs and the imported original drugs are of relatively high quality assurance, in this study, they were regarded as quality-guaranteed drugs. Analysis of the current work revealed that a tremendous upsurge was found in the overall utilization of these quality-guaranteed drugs from 57 to 94% following the policy implementation, which is consistent with the reports released by NHSA (2020) and Wang et al. (2022). Of note, the increment was more prominent in PHCs, as compared to that in the secondary and tertiary hospitals. More importantly, the utilization rate of these quality-guaranteed drugs increased consistently in all geographical regions and all ATC classes. In China, there has been a long-standing concern on the quality of generic drugs, as well as among different brands, which impeded improvement in the quality of drugs used among the general public (Tian, 2014; Chen et al., 2021). Next, fortunately, following the implementation of the “4 + 7” centralized procurement policy, the utilization rate of the quality-guaranteed drugs, at least among the “4 + 7” List drugs, remarkably improved, reflecting an overall improvement of the quality of drugs used at the population level. Meanwhile, the increase in the market share of high quality generics under the influence of the centralized procurement policy would encourage the development of the Chinese domestic pharmaceutical industry (Mao et al., 2020). In the long run, the advance of the policy may be conducive to guiding pharmaceutical enterprises to pay more attention to drug quality and innovation. In light of this, we recommend to expand the coverage of centralized procurement drugs in order to benefit more patients and, meanwhile, to get rid of the use of low-quality drugs, such as generic drugs that failed to pass the GCE assessment.

Moreover, the quantity and proportion in use of the “4 + 7” List drugs significantly increased after the policy intervention, which might be ascribed to the release of some previously unmet drug demands after drug price reduction. Besides, we found that the increment in the utilization proportion of “4 + 7” List drugs took place mostly during early months of the policy implementation, which then generally returned to the pre-intervention level. These results suggested that excessive procurement and use of related drugs might be found in medical institutions in the early stage of policy implementation, (Yang et al., 2021b), which deserves a full attention in policy monitoring in the future.

Centralized public procurement is an effective approach to redress the imbalance in pharmaceutical market leverage between supply and demand, with the logical underpinnings that the consolidation of purchasing power produces economies of scale and brings many benefits such as price reduction, improved quality assurance, rationalized choice, etc. (Huff-Rousselle, 2012). The principle for the impact of centralized procurement on hospitals’ drug utilization lies in the reshaping of the market pattern under the alliance mechanism—the influence of the centralized procurement mechanism on drug market varied by existing competition patterns, drug attributes, and buyers’ demands (Dubois et al., 2021; Wang and Zahur, 2022). Existing literature noted that centralized procurement mechanism demonstrated no limit on product choices of healthcare providers (Callea et al., 2017; Wang and Zahur, 2022), nor did we observe in this study; therefore, reasons exist to believe that changes in drug utilization may be largely derived from the increased efficiency of the procurement system and improved rationality of decision-making about drug procurement (Bandiera et al., 2008; Huff-Rousselle, 2012). In China’s current public procurement practice, our findings indeed indicated significant changes in drug utilization of policy-related drugs after the implementation of the centralized drug procurement policy, and the variations in drug use structure among healthcare settings and geographical regions were gradually diminished. Next, the promotion of overall quality of drugs used and the homogenization of drug use structure might be conducive to hierarchical diagnosis and treatment, as well as enhancement in the fairness of drug usage at population level in China.

This study demonstrates a few limitations. First, provincial procurement data (i.e., population level data), rather than the clinical medicine use of patients (such as prescriptions), were analyzed in this study. Although the research method for drug utilization is internationally accepted, the resulting DDD data cannot be followed back to the demand in individual patients. Therefore, in the future, patient- and prescriber-level research might be needed to identify the direct causes behind the observed changes in drug utilization under the “4 + 7” policy. Second, due to the lack of city-level data from non-pilot areas, in this study, we matched the control group by province (rather than by city), which might be an imperfection regarding the establishment of the control group. Also, it should be noted that the “4 + 7” pilot cities are China’s top developed areas; therefore, it is difficult to assign a control group in mainland China that completely matched the pilot group in terms of population size, economic development, medical resources, etc. In this study, we made further explorations based on our previous work (Wang et al., 2022), such as the TOPSIS matching and the stratified matching by geographical region, in an attempt to improve the comparability between intervention and control group to the greatest extent. Next, luckily, the common trends tests supported our hypothesis. Despite that, the present findings might also be confronted with the risk of bias, and one should be cautious when interpreting the results.

## Conclusion

During the 1-year contracted procurement period, the agreed procurement tasks of medical institutions were mostly well fulfilled in pilot cities, with an overall completion rate of 191.4%. After policy intervention, the drug utilization of China’s public medical institutions significantly changed, and the consumption became more concentrated to bid-winning drugs and generic drugs. Next, the variations in drug use among healthcare settings and geographical regions were gradually narrowed. Moreover, “4 + 7” centralized procurement policy significantly promoted the use proportion of quality-guaranteed drugs consistently in all regions, healthcare settings, and ATC classes. In the future, policy improvement is still needed to expand the influence coverage on drug utilization and promote equity in drug use in China.

## Data Availability

The original contributions presented in the study are included in the article/Supplementary Material, and further inquiries can be directed to the corresponding authors.

## References

[B1] Bandiera O. Prat A. Valletti T. M. (2008). Active and passive waste in government spending: Evidence from a policy experiment. SSRN J. 99 (4). 10.2139/ssrn.1115339

[B2] Callea G. Armeni P. Marsilio M. Jommi C. Tarricone R. (2017). The impact of HTA and procurement practices on the selection and prices of medical devices. Soc. Sci. Med. 174, 89–95. 10.1016/j.socscimed.2016.11.038 28013109

[B3] Chang F. (2021). Analysis on the core elements of volume-based drug procurement. Chin. Health Resour. 24 (01), 15–19. 10.13688/j.cnki.chr.2021.200774

[B4] Chen L. Yang Y. Luo M. Hu B. Yin S. Mao Z. (2020). The impacts of national centralized drug procurement policy on drug utilization and drug expenditures: The case of shenzhen, China. Int. J. Environ. Res. Public Health 17 (24), 9415. 10.3390/ijerph17249415 PMC7765443 33334027

[B5] Chen Y. Ji X. Xiao H. Unger J. M. Cai Y. Mao Z. (2021). Impact of the pilot volume-based drug purchasing policy in China: Interrupted time-series analysis with controls. Front. Pharmacol. 12, 804237. 10.3389/fphar.2021.804237 35815118 PMC9262040

[B6] Chinese Pharmaceutical Association (2020). Hospital drug use monitoring report (the first half of 2020). Available at: https://www.cpa.org.cn//?do=info&cid=75541 (accessed April 3, 2022).

[B7] Craig P. Cooper C. Gunnell D. Haw S. Lawson K. Macintyre S. (2012). Using natural experiments to evaluate population health interventions: New medical research Council guidance. J. Epidemiol. Community Health 66 (12), 1182–1186. 10.1136/jech-2011-200375 22577181 PMC3796763

[B8] Dubois P. Lefouili Y. Straub S. (2021). Pooled procurement of drugs in low and middle income countries. Eur. Econ. Rev. 132, 103655. 10.1016/j.euroecorev.2021.103655

[B56] FDA (2019). 2018: A year of advancing generic products and policies, laying the foundation for 2019. Available at: https://www.fda.gov/news-events/fda-voices/2018-year-advancing-generic-products-and-policies-laying-foundation-2019 (accessed April 14, 2022).

[B9] Fu H. (2017). Advances in China pharmaceutical policy research. Beijing: Peking Union Medical College Press.

[B10] General Office of the State Council (2019a). Notice on the pilot program of national centralized drug procurement and use (GBF [2019] No. 2). Available at: http://www.gov.cn/zhengce/content/2019-01/17/content_5358604.htm (accessed March 10, 2022).

[B11] General Office of the State Council (2019b). Opinions on strengthening the performance evaluation of tertiary public hospitals (GBF [2019] No. 4). Available at: http://www.gov.cn/zhengce/content/2019-01/30/content_5362266.htm (accessed June 17, 2022).

[B12] He J. Tang M. Cong L. Xu Y. Song J. Chen M. (2021). The impact of National Centralized Drug Procurement on the clinical management and drug use. Chin. Health Resour. 24 (01), 29–31. 10.13688/j.cnki.chr.2021.200802

[B13] Hollingworth S. Kairuz T. (2021). Measuring medicine use: Applying ATC/DDD methodology to real-world data. Pharmacy 9 (1), 60. 10.3390/pharmacy9010060 33802774 PMC8006033

[B14] Hu J. Mossialos E. (2016). Pharmaceutical pricing and reimbursement in China: When the whole is less than the sum of its parts. Health Policy 120 (5), 519–534. 10.1016/j.healthpol.2016.03.014 27080345

[B15] Hu S. (2021). The health economics theoretical basis and improvement suggestions for the national centralized drug procurement. Chin. Health Resour. 24 (1), 1223–1314. 10.13688/j.cnki.chr.2021.200942

[B16] Huang W. Zhang Z. Liu A. (2022). Differences-in-differences method to event study. Rev. Industrial Econ. 2022 (02), 1–18. 10.19313/j.cnki.cn10-1223/f.20211227.002

[B17] Huff-Rousselle M. (2012). The logical underpinnings and benefits of pooled pharmaceutical procurement: A pragmatic role for our public institutions? Soc. Sci. Med. 75 (9), 1572–1580. 10.1016/j.socscimed.2012.05.044 22835922

[B18] Joint Procurement Office (2018b). *"*4+7*" city drug centralized procurement document (No. GY-YD2018-1)* . Available at: http://www.smpaa.cn/gjsdcg/2018/11/15/8511.shtml (accessed March 10, 2022).

[B19] Joint Procurement Office (2018a). Bid-winning results of "4+7" city centralized drug procurement. Available at: http://www.smpaa.cn/gjsdcg/2018/12/17/8580.shtml (accessed March 25, 2022).

[B20] Kim S. W. Skordis-Worrall J. (2017). Can voluntary pooled procurement reduce the price of antiretroviral drugs? A case study of efavirenz. Health Policy Plan. 32 (4), 516–526. 10.1093/heapol/czw165 28052986

[B21] Li J. Zeng Y. Cao C. Wang S. Wang J. (2013). Study on differences in regional sales of Chinese medicine based on the overlap rate. China J. Pharm. Econ. 2013 (02), 25–28.

[B22] Li Z. Liu C. Zuo K. Liu J. Tang Y. (2021). Effects of volume-price contracts on pharmaceutical prices: A retrospective comparative study of public hospitals in hubei of China. Front. Pharmacol. 12, 741671. 10.3389/fphar.2021.741671 34721029 PMC8552023

[B23] Luo N. Yue J. Zhou R. Jiang B. (2022). The effects of the national drug pooled procurement (NDPP) pilot program in China. J. Chin. Pharm. Sci. 31 (3), 212–217. 10.5246/jcps.2022.03.019

[B24] Mao Z. Yang Y. Chen L. (2020). “Reform of drug supply and guarantee system in China: Policy measures and effects,” in Development report on health reform in China (2020). Editors Wang C. Liang W. (Beijing: Social Sciences Academic Press), 96–123.

[B25] NHC (2015). China drug supply information platform. Available at: http://cdsip.nhc.gov.cn/(accessed March 15, 2022).

[B26] NHC (2020). China health Statistics yearbook 2020. Beijing: Peking Union Medical College Press.

[B27] NHC (2019b). Notice on further improving the clinical equipment and use of the bid-winning drugs in the national centralized drug procurement (guoweiban yihan [2019] No. 889). Available at: http://www.nhc.gov.cn/yzygj/s7659/201912/7b1639fb14ca4cd59cd33f367455d92d.shtml (accessed January 8, 2022).

[B28] NHC (2019a). Notice on the clinical equipment and use of the bid-winning drugs in the national centralized drug procurement (guoweiban yihan [2019] No. 77). Available at: http://www.nhc.gov.cn/yzygj/s7659/201901/628ac5004d244af7ad53c0b109f0c2df.shtml (accessed January 8, 2022).

[B29] NHSA (2019b). Monitoring plan for the pilot work of national centralized drug procurement and use. Available at: http://www.nhsa.gov.cn/(accessed January 29, 2022).

[B59] NHSA (2020). Answer to reporters’ request about the second round of national centralized drug procurement and use. Available at: http://www.nhsa.gov.cn/art/2020/1/17/art_38_2264.html(accessed January 29, 2022).

[B30] NHSA (2019a). Opinions on the supporting measures of medical insurance on the pilot program of national centralized drug procurement and use ( *Yibaofa [2019] No. 18)*. Available at: http://www.nhsa.gov.cn/art/2019/3/5/art_53_1016.html (accessed January 10, 2022).

[B55] NHSA (2022). The State Council regular policy briefing: Progress on the deepen reform of centralized procurement of drug and high value medical consumables. Available at: http://www.nhsa.gov.cn/art/2022/2/11/art_14_7835.html (accessed April 14, 2022).

[B31] NMPA (2017). Catalogue of marketed drug in China. Available at: https://www.cde.org.cn/hymlj/index (accessed March 5, 2022).

[B57] Oncu S. Bayram D. Aydin V. Isli F. Aksoy M. Akici A. (2020). Knowledge, opinions and attitudes of primary care physicians about generic drugs: A cross-sectional study. Fam. Pract. 38 (3), 272–279. 10.1093/fampra/cmaa138 33340330

[B58] Qu J. Zuo W. Wang S. Du L. Liu X. Gao Y. (2021). Knowledge, perceptions and practices of pharmacists regarding generic substitution in China: A cross-sectional study. BMJ Open 11 (10), e051277. 10.1136/bmjopen-2021-051277 PMC8524276 34663661

[B32] Shi L. (2020). National drug policy and essential medicine system: Management and practice. Beijing: People's Medical Publishing House.

[B33] Tang Y. (2016). How do patients use drug use information: A research on the mechanism behind based on transparency. Wuhan: Huazhong University of Science and Technology.

[B60] Tian J. (2014). Why is there a difference in efficacy between generic drugs and original drugs?. China Drug Store 10, 14–15. 10.1136/bmjopen-2021-054346

[B34] Wang J. Yang Y. Xu L. Shen Y. Wen X. Mao L. (2022). Impact of ‘4+7’ volume-based drug procurement on the use of policy-related original and generic drugs: A natural experimental study in China. BMJ Open 12 (3), e054346. 10.1136/bmjopen-2021-054346 PMC8921850 35288385

[B35] Wang L. X. Zahur N. (2022). Procurement institutions and essential drug supply in low and middle-income countries 10.1016/j.jhealeco.2025.102996 40250027

[B36] Wang N N. Yang Y. Xu L. Mao Z. Cui D. (2021). Influence of Chinese national centralized drug procurement on the price of policy-related drugs: An interrupted time series analysis. BMC PUBLIC HEALTH 21 (1), 1883. 10.1186/s12889-021-11882-7 34663282 PMC8524972

[B37] Wang Y Y. Wang Q. Su M. Qi L. Yang L. (2021). Influence of "4+7" quantity purchase policy on the rational use of antiplatelet drugs in a hospital. China Pharm. 30 (21), 8–11. 10.3969/j.issn.1006-4931.2021.21.003

[B38] Wen X. Yin S. Cui L. Mao L. Lin Z. Yaermaimaiti Z. (2021). The effects of the national centralized drug purchasing pilot program on nucleos(t)ide analogs in shenzhen city: An interrupted time series analysis. Front. Public Health 9, 718013. 10.3389/fpubh.2021.718013 34760861 PMC8572971

[B39] WHO Collaborating Centre for Drug Statistics Methodology (2020). ATC/DDD Index 2021. Available at: https://www.whocc.no/atc_ddd_index/(accessed March 25, 2022).

[B40] WHO Collaborating Centre for Drug Statistics Methodology (2021). Guidelines for ATC classification and DDD assignment 2022. Available at: https://www.whocc.no/filearchive/publications/2022_guidelines_web.pdf (accessed March 5, 2022).

[B41] Xie J. Hu Z. Wang Y. Shao R. (2021). The influences of national centralized drug procurement policy on drug price, cost and generic drug substitution: Taking the four municipalities data. Chin. Health Econ. 40, 24–28.

[B42] Yan Y. Yan Y. (2019). A study on efficiency of health resources allocation in China. Mod. Hosp. Manag. 17 (05), 6–11. 10.3969/j.issn.1672-4232.2019.05.002

[B43] Yang Y. Chen L. Ke X. Mao Z. Zheng B. (2021b). The impacts of Chinese drug volume-based procurement policy on the use of policy-related antibiotic drugs in shenzhen, 2018-2019: An interrupted time-series analysis. BMC Health Serv. Res. 21 (1), 668. 10.1186/s12913-021-06698-5 34238290 PMC8265121

[B44] Yang Y. Geng X. Liu X. Wen X. Wu R. Cui D. (2022b). Antibiotic use in China’s public healthcare institutions during the COVID-19 pandemic: An analysis of nationwide procurement data, 2018–2020. Front. Pharmacol. 13, 813213. 10.3389/fphar.2022.813213 35237164 PMC8882946

[B45] Yang Y. Hu R. Geng X. Mao L. Wen X. Wang Z. (2022a). The impact of National Centralised Drug Procurement policy on the use of policy-related original and generic drugs in China. Int. J. Health Plann. Manage. 37, 1650–1662. 10.1002/hpm.3429 35132676

[B46] Yang Y. Tong R. Yin S. Mao L. Xu L. Hao S. (2021a). The impact of "4+7" volume-based drug procurement on the volume, expenditures, and daily costs of antihypertensive drugs in shenzhen, China: An interrupted time series analysis. BMC Health Serv. Res. 21 (1), 1275. 10.1186/s12913-021-07143-3 34823516 PMC8620621

[B47] Yi H. Miller G. Zhang L. Li S. Rozelle S. (2015). Intended and unintended consequences of China's zero markup drug policy. Health Aff. 34 (8), 1391–1398. 10.1377/hlthaff.2014.1114 26240254

[B48] Yip W. C. Hsiao W. C. Chen W. Hu S. Ma J. Maynard A. (2012). Early appraisal of China's huge and complex health-care reforms. Lancet 379 (9818), 833–842. 10.1016/S0140-6736(11)61880-1 22386036

[B49] Yuan J. Lu Z. K. Xiong X. Jiang B. (2021). Lowering drug prices and enhancing pharmaceutical affordability: An analysis of the national volume-based procurement (NVBP) effect in China. BMJ Glob. Health 6 (9), e005519. 10.1136/bmjgh-2021-005519 PMC8438819 34518200

[B54] Yu C. (2020). The practical effects and system concerns of "4+7" drug procurement.. Journal of Southwest Minzu University (Humanities and Social Science) 41 (04), 34–39. 10.3969/j.issn.1004-3926.2020.04.006

[B50] Zeng W. (2013). A price and use comparison of generic versus originator cardiovascular medicines: A hospital study in chongqing, China. BMC Health Serv. Res. 13 (1), 390. 10.1186/1472-6963-13-390 24093493 PMC3851002

[B51] Zeng W. Zhen J. Feng M. Campbell S. M. Finlayson A. E. Godman B. (2014). Analysis of the influence of recent reforms in China: Cardiovascular and cerebrovascular medicines as a case history to provide future direction. J. Comp. Eff. Res. 3 (4), 371–386. 10.2217/cer.14.28 25275234

[B52] Zhang M. Zhang Z. (2015). Differences of Chinese herbs used in influenza between South and North areas of China. Guid. J. Traditional Chin. Med. 21 (19), 13–15+22. 10.13862/j.cnki.cn43-1446/r.2015.19.005

[B53] Zhang Y. Ma Q. Chen Y. Gao H. (2017). Effects of public hospital reform on inpatient expenditures in rural China. Health Econ. 26 (4), 421–430. 10.1002/hec.3320 26842555

