# Supplementary material for: The change of drug utilization in China’s public healthcare institutions under the “4 + 7” centralized drug procurement policy: Evidence from a natural experiment in China
Source: Front Pharmacol. 2022 Aug 23;13:923209. doi: 10.3389/fphar.2022.923209 (PMC9445493; doi:10.3389/fphar.2022.923209)
Supplement: Supplementary file 1 [file Table1.DOCX]

Supplementary Material

## Table S1. Results of (unweighted) TOPSIS.

| Regions | | Per capita GDP (CNY) | Population size (10,000) | Number of health institutions (per 1000 population) | Number of hospital beds (per 1000 population) | Number of skilled health workers (per 1000 population) | Number of licensed (assistant) doctors (per 1000 population) | Per capital health expenditure (CNY) | Annual average clinical visits | Annual hospitalization rate (%) | TOPSIS  Score |
| --- | --- | --- | --- | --- | --- | --- | --- | --- | --- | --- | --- |
| East China | Xiamen* | 142739.00 | 429.00 | 0.49 | 4.38 | 8.61 | 3.54 | 4276.70 | 6.27 | 15.30 | 0.1665 |
|  | Shanghai* | 157279.00 | 2428.00 | 0.23 | 6.03 | 8.40 | 3.10 | 10430.53 | 11.35 | 18.70 | 0.3228 |
|  | Jiangsu⟊ | 123607.00 | 8070.00 | 0.43 | 6.39 | 7.80 | 3.20 | 5525.61 | 7.65 | 18.90 | 0.2546 |
|  | Zhejiang⟊ | 107624.00 | 5850.00 | 0.58 | 5.99 | 8.90 | 3.50 | 5881.25 | 11.65 | 18.90 | 0.2856 |
| North China | Tianjin* | 90371.00 | 1562.00 | 0.38 | 4.37 | 7.00 | 3.00 | 6233.15 | 7.87 | 10.90 | 0.1338 |
|  | Beijing* | 164220.00 | 2154.00 | 0.48 | 5.93 | 12.60 | 4.90 | 13766.77 | 11.56 | 17.90 | 0.6110 |
|  | Inner Mongolia ⟊ | 67852.00 | 2540.00 | 0.97 | 6.34 | 7.70 | 3.10 | 4604.72 | 4.21 | 14.30 | 0.1526 |
|  | Shanxi⟊ | 45724.00 | 3729.00 | 1.13 | 5.86 | 6.90 | 2.80 | 3465.29 | 3.53 | 13.40 | 0.1550 |
| Central China | Guangzhou* | 131400.00 | 1831.21 | 0.28 | 5.47 | 9.18 | 3.20 | 5317.60 | 7.74 | 15.80 | 0.1797 |
|  | Shenzhen* | 159883.00 | 1710.40 | 0.29 | 2.81 | 6.01 | 2.36 | 5317.60 | 7.74 | 15.80 | 0.1868 |
|  | Hubei⟊ | 77387.00 | 5927.00 | 0.60 | 6.80 | 7.00 | 2.60 | 4354.57 | 5.97 | 23.10 | 0.1897 |
|  | Hunan⟊ | 57540.00 | 6918.00 | 0.83 | 7.32 | 7.30 | 2.80 | 4006.25 | 4.06 | 23.40 | 0.2056 |
| Northeast China | Dalian* | 99996.00 | 598.70 | 0.69 | 8.22 | 9.58 | 3.85 | 4160.65 | 4.59 | 16.30 | 0.1639 |
|  | Shenyang* | 77777.00 | 755.40 | 0.69 | 9.52 | 10.82 | 4.21 | 4160.65 | 4.59 | 16.30 | 0.1801 |
|  | Jilin⟊ | 43475.00 | 2691.00 | 0.82 | 6.33 | 7.00 | 2.90 | 4356.76 | 4.10 | 15.00 | 0.1258 |
|  | Heilongjiang⟊ | 36183.00 | 3751.00 | 0.54 | 7.00 | 6.30 | 2.50 | 4027.08 | 3.00 | 16.10 | 0.1180 |
| Southwest China | Chengdu* | 103386.00 | 1658.10 | 0.73 | 8.98 | 11.16 | 4.13 | 4424.37 | 6.69 | 23.70 | 0.2146 |
|  | Chongqing* | 75828.00 | 3124.00 | 0.67 | 7.42 | 7.20 | 2.70 | 4530.36 | 5.62 | 24.10 | 0.1612 |
|  | Guizhou⟊ | 46433.00 | 3623.00 | 0.79 | 7.31 | 7.40 | 2.50 | 3838.96 | 4.85 | 23.70 | 0.1653 |
|  | Tibet⟊ | 48902.00 | 351.00 | 1.98 | 4.87 | 6.00 | 2.70 | 5395.01 | 4.66 | 8.70 | 0.4261 |
| Northwest China | Xi'an* | 92256.00 | 1020.35 | 0.69 | 7.11 | 11.00 | 10.65 | 4706.80 | 5.39 | 21.10 | 0.4569 |
|  | Qinghai⟊ | 48981.00 | 608.00 | 1.07 | 6.82 | 7.80 | 2.90 | 5843.23 | 4.38 | 17.40 | 0.4978 |

Note: * “4+7” Pilot regions; ⟊ selected control regions. GDP, gross domestic product; CNY, Chinese yuan; TOPSIS, technique for order performance by similarity to ideal solution.

## Table S2. The name of drugs included in this study.

| No. | “4+7” List drugs | Alternative drugs |
| --- | --- | --- |
| 1 | Atorvastatin | Pitavastatin, Pravastatin, Fluvastatin, Simvastatin, Lovastatin, Ezetimibe and Simvastatin, Xuezhikan, Zhibituo, Zhibitai |
| 2 | Rosuvastatin | Pitavastatin, Pravastatin, Fluvastatin, Simvastatin, Lovastatin, Ezetimibe and Simvastatin, Xuezhikan, Zhibituo, Zhibitai |
| 3 | Clopidogrel | Ticagrelor, Aspirin, Tiolopidine, Cilostazol |
| 4 | Irbesartan | Olmesartan, Candesartan, Valsartan, Telmisartan, Losartan, Allisartan, Olmesartan and Amlodipine, Valsartan and Amlodipine, Olmesartan and Hydrochlorothiazide, Losartan and Hydrochlorothiazide, Telmisartan and Hydrochlorothiazide, Valsartan and Hydrochlorothiazide, Irbesartan and Hydrochlorothiazide, Candesartan and Hydrochlorothiazide |
| 5 | Amlodipine | Levoamlodipine, Amlodipine folate, Felodipine, Nifedipine, Amlodipine and Atorvastatin, Amlodipine and Benazepril, Olmesartan and Amlodipine, Valsartan and Amlodipine |
| 6 | Entecavir | Tenofovir Disoproxil, Tenofovir Alafenamide, Lamivudine, Adefovir, Telbivudine |
| 7 | Escitalopram | Citalopram, Fluoxetine, Paroxetine, Votioxetine, Duloxetine, Sertraline, Fluvoxamine, Bupropion, Trazodone, Venlafaxine |
| 8 | Paroxetine | Fluoxetine, Votioxetine, Duloxetine, Escitalopram, Citalopram, Sertraline, Fluvoxamine, Bupropion, Trazodone, Venlafaxine |
| 9 | Olanzapine | Paliperidone, Clozapine, Aripirazole, Metamizole and Chlorpromazine, Amisulpride, Loxapine, Haloperidol, Quetiapine, Fluphenazine Decanoate, Chlorpromazine, Ziprasidone, Trifluoperazine, Ziprasidone |
| 10 | Cefuroxime | Cefaclor, Cefalexin, Cefprozil, Cefdinir, Cefmnoxime, Cefixime, Cefadroxil |
| 11 | Risperidone | Quetiapine, Aripirazole, Metamizole and Chlorpromazine, Amisulpride, Loxapine, Haloperidol, Quetiapine, Fluphenazine Decanoate, Chlorpromazine, Ziprasidone, Trifluoperazine, Ziprasidone, Perphenazine, Sulpiride, Penfluridol, Piperothiazide, Piperothiazide, Tiapride |
| 12 | Gefitinib | Icotinib, Erlotinib, Afatinib, Osimertinib |
| 13 | Fosinopril | Captopril, Benazepril, Perindopril, Ramipril, Amlodipine and Benazepril, Benazepril and Hydrochlorothiazide, Compound Captopril, Lisinopril and Hydrochlorothiazide, Enalapril folate, Perindopril and Indapamide, Perindopril and Amlodipine |
| 14 | Irbesartan and Hydrochlorothiazide | Olmesartan and Hydrochlorothiazide, Losartan and Hydrochlorothiazide, Telmisartan and Hydrochlorothiazide, Valsartan and Hydrochlorothiazide, Candesartan and Hydrochlorothiazide, Olmesartan, Candesartan, Valsartan, Telmisartan, Allisartan, Olmesartan and Amlodipine, Valsartan and Amlodipine |
| 15 | Lisinopril | Captopril, Benazepril, Enalapril, Perindopril, Ramipril, Amlodipine and Benazepril, Benazepril and Hydrochlorothiazide, Compound Captopril, Lisinopril and Hydrochlorothiazide, Enalapril folate, Perindopril and Indapamide, Perindopril and Amlodipine |
| 16 | Tenofovir Disoproxil | Tenofovir Alafenamide, Lamivudine, Adefovir, Telbivudine |
| 17 | Losartan | Candesartan, Valsartan, Telmisartan, Olmesartan, Allisartan, Olmesartan and Amlodipine, Valsartan and Amlodipine, Olmesartan and Hydrochlorothiazide, Losartan and Hydrochlorothiazide, Telmisartan and Hydrochlorothiazide, Valsartan and Hydrochlorothiazide, Candesartan and Hydrochlorothiazide |
| 18 | Enalapril | Captopril, Benazepril, Perindopril, Ramipril, Amlodipine and Benazepril, Benazepril and Hydrochlorothiazide, Compound Captopril, Lisinopril and Hydrochlorothiazide, Enalapril folate, Perindopril and Indapamide, Perindopril and Amlodipine |
| 19 | Levetiracetam | Oxcarbazepine, Magnesium Valproate, Valproate Sodium, Carbamazepine, Lamotrigine, Topiramate |
| 20 | Imatinib | Nilotinib, Dasatinib |
| 21 | Montelukast | Pemirolast , Pranlukast, Seratrodast, Ibudilast, Zafirlukast |
| 22 | Montmorilonite | Berberine, Loperamide, Albumini Tannas |
| 23 | Pemetrexed | - |
| 24 | Flurbiprofen | Parecoxib, Piroxicam, Diclofenac, Tromethamine, Indomethacin |
| 25 | Dexmedetomidine | Midazolam, Diclofenac |

## Figure S1. Results of common pre-trend tests for DID.


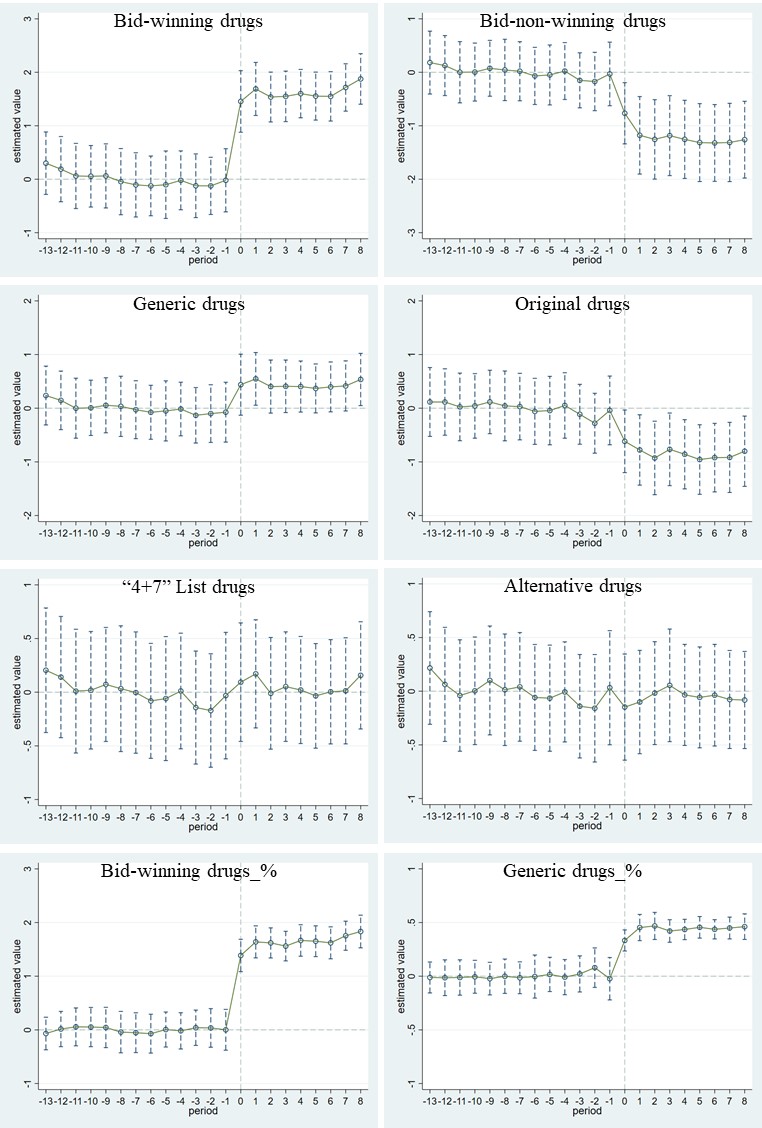


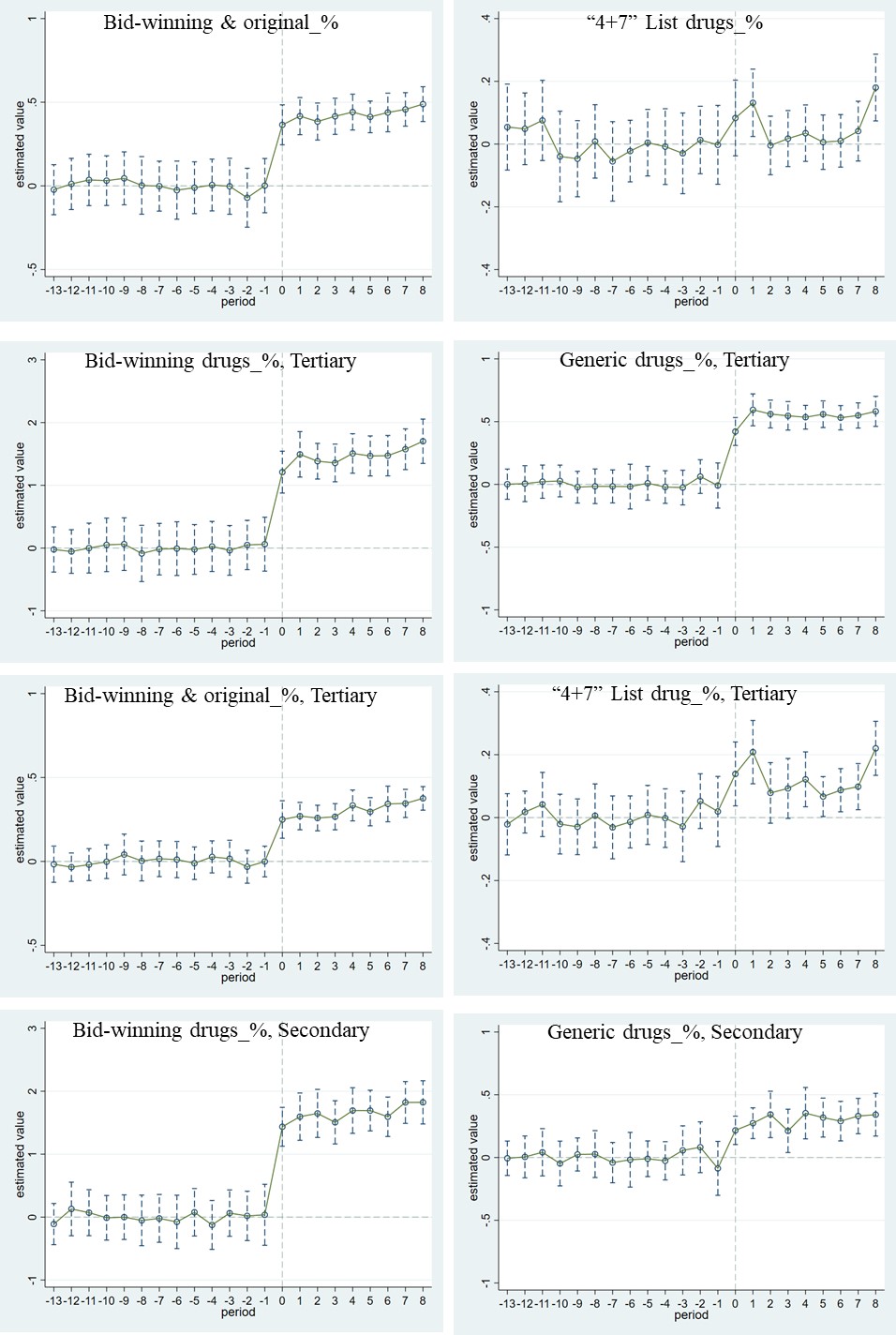


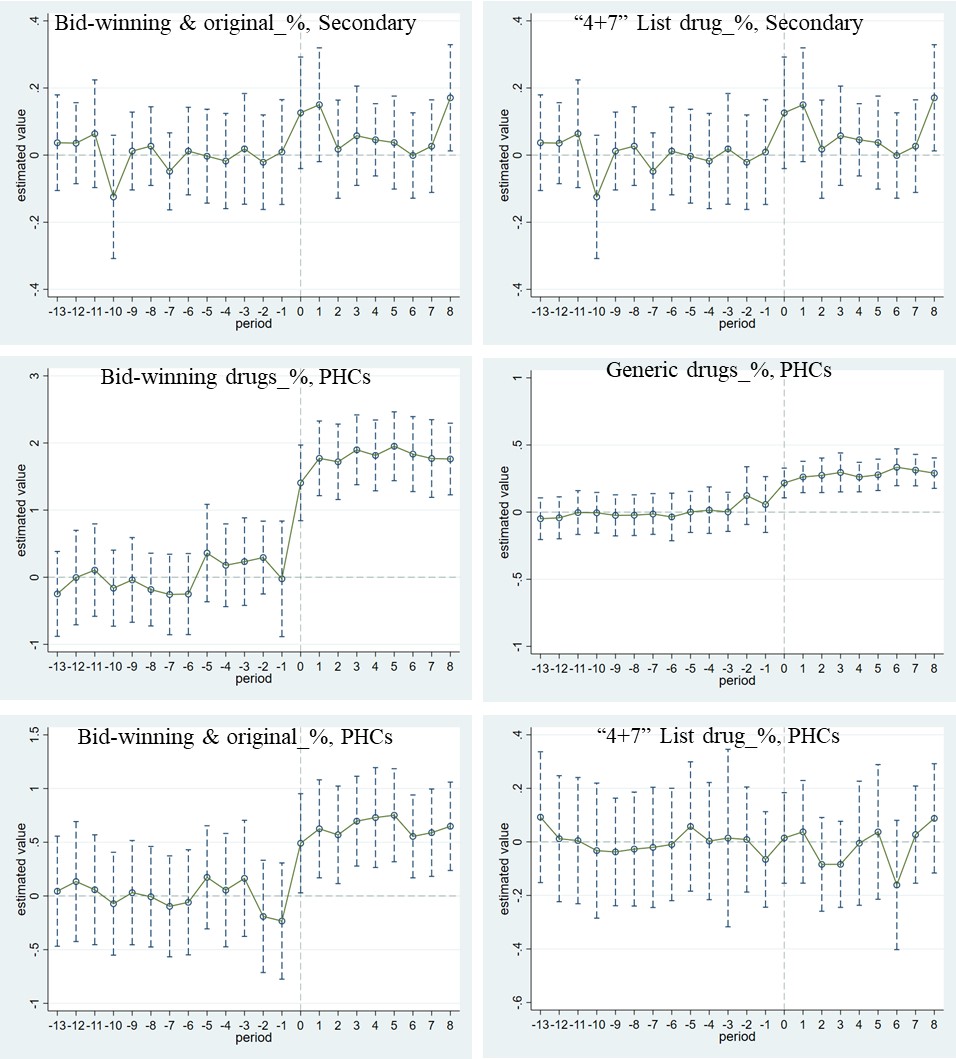


Note: The graphs graphically shows the point estimates and 95% CIs for the interaction terms between a series of dummy variables (indicating each month), and the other dummy variables (indicating the intervention group). It the assumption of common pre-trends holds, the estimations in the pre-intervention periods will not be different from zero.
